# ﻿Descriptions of four new species of atyid shrimp (Crustacea, Decapoda, Atyidae) in Vietnam

**DOI:** 10.3897/zookeys.1247.148607

**Published:** 2025-07-28

**Authors:** Van Tu Do, Thi Yen Phan, Kristina von Rintelen, Hung Anh Le, Thomas von Rintelen

**Affiliations:** 1 Institute of Biology, Vietnam Academy of Science and Technology, 18 Hoang Quoc Viet, Ha Noi, Vietnam Graduate University of Science and Technology, Vietnam Academy of Science and Technology Ha Noi Vietnam; 2 Graduate University of Science and Technology, Vietnam Academy of Science and Technology, 18 Hoang Quoc Viet, Ha Noi, Vietnam Institute of Biology, Vietnam Academy of Science and Technology Ha Noi Vietnam; 3 Hung Vuong University, Nong Trang Ward, Viet Tri City, Vietnam Hung Vuong University Viet Tri City Vietnam; 4 Museum für Naturkunde, Leibniz Institute for Evolution and Biodiversity Science, Berlin, Germany Museum für Naturkunde, Leibniz Institute for Evolution and Biodiversity Science Berlin Germany

**Keywords:** *Caridina*, distribution, diversity, endemism, freshwater fauna, taxonomy

## Abstract

Recent surveys revealed a high biodiversity of freshwater shrimps, Atyidae De Haan, 1849 in northern Vietnam. Here, we describe four new species of the genus *Caridina*, *C.ngocson***sp. nov.**, *C.xuanlien***sp. nov.**, *C.tanson***sp. nov.**, and *C.tamkim***sp. nov**. The new species can be distinguished from their congeners by distinct characteristics of the rostrum, stylocerite, first and second pereiopods, male first and second pleopods, and uropodal diaeresis. Molecular phylogenetic analyses of the mitochondrial cytochrome oxidase subunit I (COI) and 16S ribosomal RNA (16S) genes support the distinctness of the new species from all other species examined.

## ﻿Introduction

Northern Vietnam is a transition zone between the Palearctic fauna of mainland China and the Southeast Asian, extending as far south as Malaysia and Indonesia. Such transitional zones are often rich in species, and northern Vietnam is no exception. Its humid climate and varied topography and geology have contributed to the local wealth of biodiversity (Vermeulen and Maassen 2003). Data from previous studies on biodiversity in Vietnam have shown that northern Vietnam, especially the northeast, has a high level of diversity and endemism. The research on freshwater invertebrates such as crabs, mussels, snails, and shrimps have shown a remarkable level of species diversity in this area (Köhler et al. 2012; Do et al. 2017, 2018).

Among freshwater shrimp families, the Atyidae are the most diverse group (De Grave et al. 2008). The history of atyid studies in Vietnam was presented in Do et al. (2020). Prior to this study, a total of 26 atyid species were recorded from Vietnam (Bouvier 1904, 1919, 1925; Dang 1967, 1975; Dang et al. 1980; Cai et al. 1999; Nguyen 1999; Li and Liang 2002; Dang and Do 2007a, 2007b; Do and Dang 2010; Dang and Ho 2012; Do et al. 2020, 2021a, 2021b, 2021c; Phan et al. 2021a; Phan et al. 2021b). This is a very small number compared to the neighboring fauna of China with 147 species (Jin et al. 2021). The study of specimens of *Caridina* H. Milne Edwards, 1837 (Milne Edwards 1837), collected in the forested mountainous areas of northern Vietnam revealed four previously undescribed species. These new species can be distinguished from all congeners by several morphological characters and their mitochondrial DNA sequences, cytochrome oxidase subunit I (COI) and 16S ribosomal RNA (16S) genes. The aim of the present study was to describe these new species.

## ﻿Materials and methods

**Figure 1.**
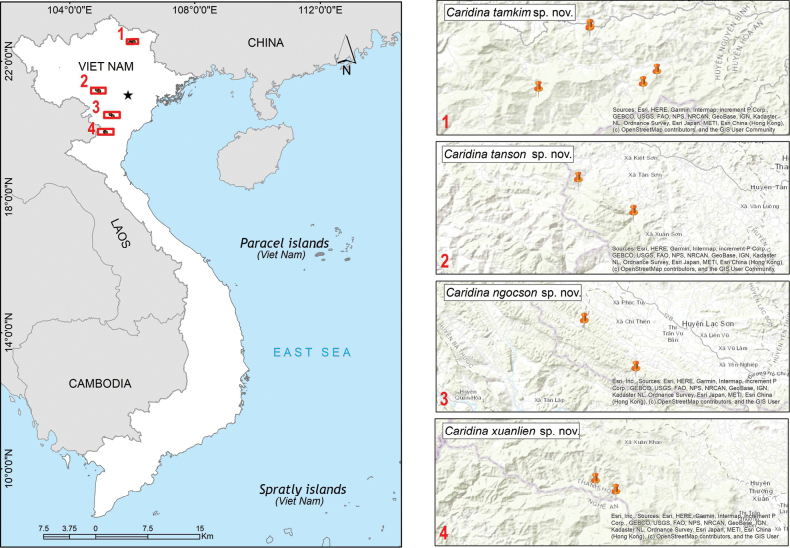
Collection sites of the new species.

Specimens of the new species were collected by hand nets from streams in Cao Bang, Phu Tho, Hoa Binh, and Thanh Hoa provinces, northern Vietnam (Fig. 1). After collection, living specimens were photographed with a Nikon D5600 digital camera to record the coloration, and then preserved in 70–95% ethanol. In the laboratory, the specimens were dissected, and the parts of the body were photographed under a Nikon SMZ18 stereo microscope. The drawings were then processed with Adobe Photoshop CS5 and Adobe Illustrator CS2 graphics software. The map was produced using ArcGIS version 10.8. All photographic materials presented in this paper were captured by Do VT. The material examined was deposited at the Institute of Biology (**IB**), Vietnam Academy of Science and Technology, Hanoi, Vietnam, and at the crustacean wet collection at the Museum für Naturkunde (**ZMB**), Berlin, Germany. The holotypes are deposited at the IB. All terminology follows Chace (1997). The abbreviation **cl** is used for carapace length, measured from the postorbital margin to the posterior median margin of the carapace in mm. The rostral formula refers to the number of dorsal teeth on the carapace posterior to the orbital margin + number of dorsal teeth on the rostrum anterior to the orbital margin/number of ventral teeth of the rostrum.

For the molecular analyses, DNA was purified from ~2 mm^3^ of abdominal muscle tissue with a Qiagen BioSprint 96 using the manufacturer’s protocol. Polymerase chain reaction (PCR) was used to amplify two mitochondrial gene fragments, a ~590 bp region of the 16S ribosomal RNA gene (16S) using primers 16S-F-Car and 16S-R-Car1, and a 861 bp fragment of the Cytochrome Oxidase subunit I gene (COI) using primers COI-F-Car and COI-R-Car (von Rintelen et al. 2007). Fragments of the mitochondrial 16S and COI were sequenced using primers 16S-F-Car and 16S-R-Car1 (16S), and COI-F-Car and COI-R-Car (COI) (von Rintelen et al. 2007), or, for COI only, COI-F-Car and COI-R-H16mod3 (1087 bp fragment extending COI-F-Car/COI-R-Car fragment at 3’ end; 5’ CAAYKATCTGCCATTTTAGA), sometimes in combination with COI-F-Car and COI-R-int (458 bp fragment at 5’ end of COI-F-Car/COI-R-Car fragment; 5’ GCAA-TAATTATAGTTGCTGA). In the latter case, sequencing was done using COI-R-int and COI-R-H16mod3.

PCR was performed in 25 µl volumes containing 1x Taq buffer, 1.5 mM MgCl2, 200 µM each dNTP, 1 U Taq polymerase, ca. 50–100 ng DNA, and ddH2O. After an initial denaturation step of 3 min at 94 °C, cycling conditions were 35 cycles at 94 °C for 35 s, 45 °C (COI) or 50 °C (16S) for 60 s, and 72 °C for 180 s (COI) or 90 s (16S), with a final elongation step of 5 min at 72 °C. The same primers were used in PCR and sequencing. PCR products were sent to Macrogen Europe for purification and cycle sequencing of both strands of each gene.

Contigs of forward and reverse strands were assembled using Geneious Prime (v. 2021.1.1) and corrected by eye. Sequences were aligned by eye (COI) and with MAFFT (16S) (Katoh and Standley 2013) as implemented in Geneious. In addition to the sequences generated for this study, sequences of most other Vietnamese atyid species and additional sequences of three species from outside Vietnam published by Do et al. (2020, 2021a, 2021b) plus five outgroup species from the genera *Atya*, *Atyopsis*, and *Potimirim* were included in the analysis (Do et al. 2020, 2021a, 2021b). The resulting alignments had a length of 821 bp (COI) and 545 bp (16S). To determine the best substitution model for Bayesian inference analyses (see below), hierarchical likelihood ratio tests were conducted with jModelTest (Posada 2008) on both sequence sets (24 models tested). Based on both the Akaike Information Criterion, the GTR + I + G and HKY + I + G models were chosen for COI and 16S, respectively, which were subsequently analyzed concatenated. All new sequences have been deposited in GenBank (see Table 1).

**Table 1.** Specimens of atyid taxa used for DNA sequencing.

Species	Museum acc. no.	Locality	GenBank acc. no.	
	(DNA lab code)		COI	16S
*Atyagabonensis*	MfN (DNA Crust 437)	West Africa (pet trade)	EF489962	EF489989
*Atyascabra*	MfN (DNA Crust 510)	Panama (pet trade)	EF489964	EF489985
*Atyopsismoluccensis*	MfN (DNA Crust 222)	Indo Pacific region (pet shop in Germany)	DQ681246	DQ681281
*Atyopsisspinipes*	ZMB 29779 (779)	Indonesia, Aceh, Aceh Selatan	PV644212	PV642627
*Caridinacaobangensis*	ZMB 30255 (1942)	Vietnam, Cao Bang, Ha Quang, Truong Ha, Pac Bo	MT526826	MT526809
*Caridinacantonensis*	ZMB 32183 (669)	China, Zuhai	KP168758	KP168717
*Caridinaclinata*	ZMB 31777 (2511)	Vietnam, Ninh Binh, Cuc Phuong	MT526827	MT526810
*Caridinacucphuongensis*	ZMB 30234 (2093)	Vietnam, Ninh Binh, Cuc Phuong	PV644218	PV642634
	ZMB 31744 (2503)		MT526828	MT526811
*Caridinagracilipes*	ZMB 30231 (2089)	Vietnam, Hai Phong, Thuy Nguyen	MT526829	MT526812
*Caridinahaivanensis*	ZMB 30304-1 (2042)	Vietnam, Thua Thien-Hue, Hai Van	PV644215	PV642631
	ZMB 30304-2 (2043)		PV644216	PV642632
*Caridinalanceifrons*	ZMB 29638 (1954)	Vietnam, Hoa Binh, Da Bac	MT526831	MT526814
*Caridinamacrophora*	ZMB 30263 (1958)	Vietnam, Hai Phong, Thuy Nguyen	MT526832	MT52681
*Caridinanamdat*	ZMB 30341-3 (2540)	Vietnam, Bac Kan, Cho Moi, Tan Son, Nam Dat	MZ484397	MZ484401
	ZMB 30341-4 (2541)		MZ484398	MZ484402
	ZMB 30342 (2543)		MZ484399	MZ484403
*Caridinangocson* sp. nov.	ZMB 30276-1 (1984)	Vietnam, Hoa Binh, Lac Son, Ngoc Son	PV644213	PV642628
	ZMB 30276-2 (1985)		PV644214	PV642629
*Caridinanguyeni*	ZMB 30280 (1993)	Vietnam, Cao Bang, Ha Quang, Truong Ha, Pac Bo	MT526833	MT526816
*Caridinapacbo*	ZMB 30295 (2023)	Vietnam, Cao Bang, Ha Quang, Truong Ha, Pac Bo	MT526835	MW525213
*Caridinapeninsularis*	ZMB 29341 (391)	Malaysia, Sarawak, Matan	PV644211	MN399187
*Caridinapseudoserrata*	ZMB 30343 (2544)	Vietnam, Cao Bang, Quang Yen, Tu Do	MT52683	MT526818
*Caridinarubropunctata*	ZMB 30314 (2061)	Vietnam, Thai Nguyen, Dong Hy, Van Lang	MT526838	MT526819
*Caridinaserrata*	ZMB 30306 (2047)	Vietnam, Quang Nam, Cu Lao Cham Island	PV644217	PV642633
	ZMB 32189 (671)	China, Hong Kong, Hong Kong Island	KP168722	KP168793
*Caridinatamkim* sp. nov.	ZMB 32923 (3556)	Vietnam, Nguyen Binh District, Tam Kim	PV644225	PV642642
	ZMB 32924-1 (3553)	Vietnam, Cao Bang, Nguyen Binh, Tam Kim	PV644224	PV642641
	ZMB 32924-2 (3555)		PV644223	PV642640
	ZMB 33788 (3549)	Vietnam, Cao Bang, Nguyen Binh	PV644222	PV642639
	ZMB 33814-2 (3627)		PV644226	PV642643
*Caridinatanson* sp. nov.	ZMB 32979-1 (3752)	Vietnam, Phu Tho, Tan Son, Dong Son	PV644229	PV642646
	ZMB 32979-2 (3753)		PV644230	PV642647
*Caridinathachlam*	ZMB 30338 (2533)	Vietnam, Thanh Hoa, Thach Thanh, Thach Lam	MW505997	MW505991
	ZMB 31773 (2529)		MW505999	MW505993
	ZMB 31781 (2519)	Vietnam, Ninh Binh, Nho Quan, Cuc Phuong	MW506000	MW505994
*Caridinatricincta*	ZMB 30360-1 (2572)	Vietnam, Ha Giang, Bac Me, Lac Nong	MT526839	MT526822
	ZMB 30360-2 (2573)		MT526840	MT526823
	ZMB 30363-1 (2576)		MT526841	MT526824
*Caridinaxuanlien* sp. nov.	ZMB 32944-1 (3529)	Vietnam, Thanh Hoa, Thuong Xuan, Van Xuan	PV644220	PV642637
	ZMB 32944-2 (3530)		PV644221	PV642638
	ZMB 32948-1 (3665)	Vietnam, Thanh Hoa, Thuong Xuan, Yen Nhan	PV644227	PV642644
	ZMB 32948-2 (3668)		PV644228	PV642645
*Neocaridinapalmata*	ZMB 30256 (1944)	Vietnam, Cao Bang, Ha Quang, Truong Ha, Pac Ma	MT526843	MT526825
*Paracaridinazijinica*	ZMB 32180 (663)	China, Heyuan	KP168798	KP168782
*Potimirimpotimirim*	ZMB 29476 (431)	Panama, Bocas del Toro	EF489975	FN995386

Phylogenetic trees were reconstructed by Bayesian inference (Huelsenbeck and Ronquist 2001) using MrBayes 3.2.6 (Ronquist et al. 2012). The MCMCMC-algorithm was run with four independent chains for 10,000,000 generations, samplefreq = 500, and burnin = 25%. Maximum likelihood (ML) analyses were run with IQ-TREE (Nguyen et al. 2015) using W-IQ-TREE (Trifinopoulos et al. 2016), and branch support was obtained through the implemented ultrafast bootstrap (10,000 replicates (Hoang et al. 2018)). The BI and ML analyses were run using two gene partitions with the models specified above (BI) and estimated in W-IQ-TREE: COI – TIM2+F+I+4; 16S – TPM3u+F+I+G (ML). Genetic distances were calculated using MEGA 11 (Stecher et al. 2020; Tamura et al. 2021).

## ﻿Taxonomy

### ﻿Family Atyidae De Haan, 1849

**Genus *Caridina* H. Milne Edwards, 1837**

#### ﻿ Caridina ngocson sp. nov.

Taxon classificationAnimaliaDecapodaAtyidae

9F18B6F6-A355-5E5F-B488-68BB80DC570C

https://zoobank.org/59EB6C32-0516-4725-AAC5-523AAE16BA4A

Figs 2 , 3 , 4

##### Type material.

***Holotype*.** • Adult male, cl 4.6 mm, IB-FS 007, Vietnam, Hoa Binh Province, Lac Son District, Ngoc Son Commune, Van Village, a small stream, 20°27'55.721"N, 105°18'8.027"E, 23 March 2017, collected by Van Tu Do. ***Paratypes*.** • 7 males, cl 3.8–5.3 mm, 4 females, cl 4.2–5.3 mm, 50 additional specimens, ZMB 30276, same data as holotype; • male, cl 4.4, female, cl 5 mm, IB-FS 008, Vietnam, Hoa Binh Province, Lac Son District, Ngoc Son Commune, a small stream, 20°24'11.92"N, 105°22'23.5"E, collected by Thi Yen Phan, 02 February 2023.

##### Comparative material.

*Caridinaserrata* Stimpson, 1860. • 10 males, cl 3.1–4.7, 5 females, cl 4.0–4.6, ZMB 30306, Vietnam, Quang Nam Province, Hoi An City, Cu Lao Cham Island, a small stream running into the reservoir, 15°56'34.9"N 108°31'22.7"E, collected by Pham The Cuong, 10 May 2017.

##### Diagnosis.

**Figure 2.**
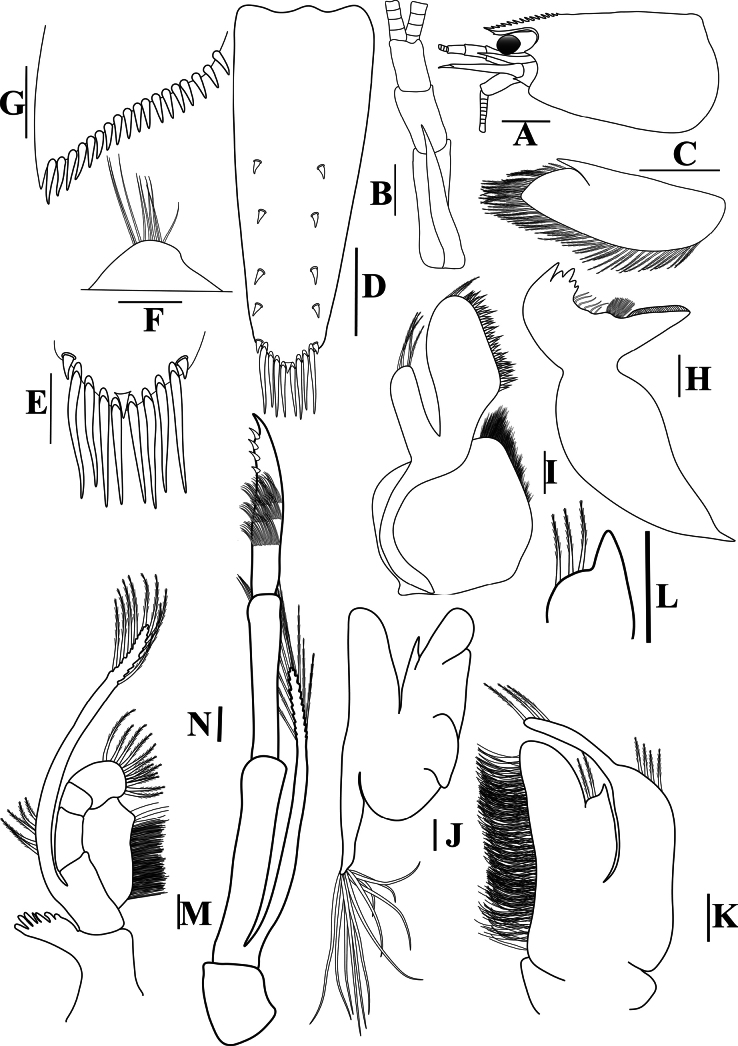
*Caridinangocson* sp. nov. adult male, cl 4.6 mm (ZMB 30276). **A.** Cephalothorax and cephalic appendages, lateral view; **B.** Antennular peduncle; **C.** Scaphocerite; **D.** Telson; **E.** Distal portion of telson; **F.** Preanal carina; **G.** Uropodal diaeresis; **H.** Mandible; **I.** Maxillula; **J.** Maxilla; **K.** First maxilliped; **L.** Distal end of palp of first maxilliped; **M.** Second maxilliped; **N.** Third maxilliped. Scale bars: 1 mm (**A**); 0.5 mm (**B–D**); 0.2 mm (**E–J**).

**Figure 3.**
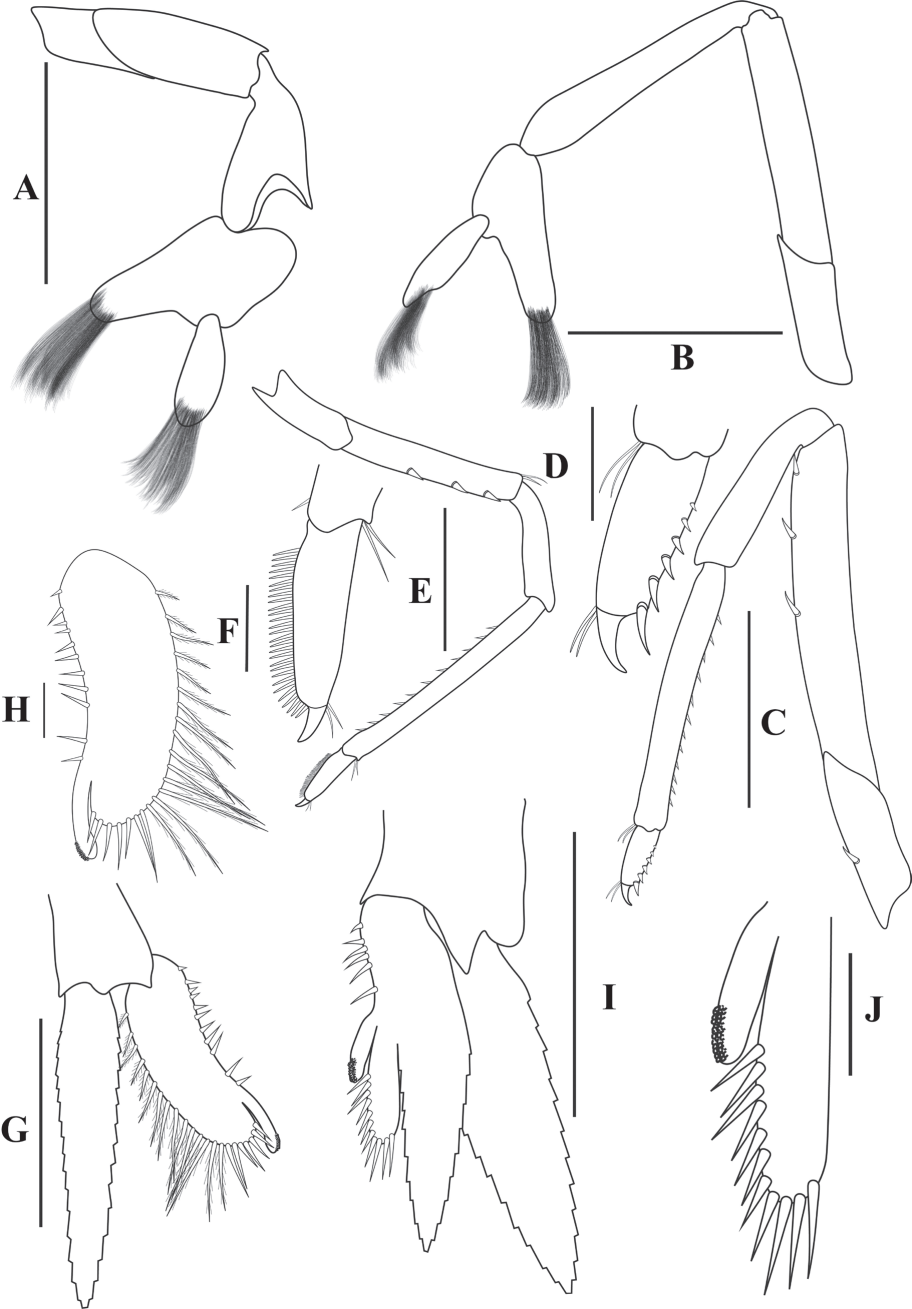
*Caridinangocson* sp. nov. adult male, cl 4.6 mm (ZMB 30276). **A.** First pereiopod; **B.** Second pereiopod; **C.** Third pereiopod; **D.** The same, dactylus; **E.** Fifth pereiopod; **F.** The same, dactylus; **G.** Male first pleopod; **H.** Endopod of male first pleopod; **I.** Male second pleopod; **J.** Appendix masculina and interna of male second pleopod. Scale bars: 1 mm (**A, B, C, F, G, I**); 0.2 mm (**D, E, H, J**).

*Caridinangocson* sp. nov. can be distinguished from other members of the genus *Caridina* by a combination of the following characteristics: short, slender, and slightly downward-curving rostrum, reaching from beginning to middle of second segment of antennular peduncle (Fig. 2A); stylocerite extending beyond distal end of basal antennular peduncle; rostral formula of 4–6+5–11/0–2; well-developed eyes (Fig. 2B); kidney-shaped endopod of male first pleopod, with appendix interna slightly exceeding terminal margin of endopod by 0.23 of its length (Fig. 3G, H); appendix masculina of male second pleopod slender, reaching approximately 0.43× endopod length; appendix interna extending ~0.60× length of appendix masculina (Fig. 3I, J); and large eggs.

##### Description.

***Cephalothorax and cephalic appendages*.** Carapace length 3.8–5.3 mm (median 4.4 mm, *n* = 12). Rostrum short and slender, straight, reaching to beginning to middle of second segment of antennular peduncle, 0.25–0.4 (median 0.33) × as long as carapace, rostral formula 4–6+5–11/0–2 (Fig. 2A). Suborbital angle acute, completely fused with antennal spine; pterygostomian margin rounded, slightly introduced forward (Fig. 2A). Eyes well developed with globular cornea, anterior end reaching to 0.8× length of basal segment of antennular peduncle (Fig. 2A). Antennular peduncle 0.47–0.58 (median 0.54) × as long as carapace; basal segment 1.98–2.39 (median 2.05) × as long as second segment, second segment 0.96–1.24 (median 1.05) × as long as third segment (Fig. 2B). Stylocerite reaching to beginning or to 0.4× length of second segment of antennular peduncle (Fig. 2B). Scaphocerite ovate, reaching beyond distal end of antennular peduncle, 2.55–2.97 (median 2.55) × as long as wide (Fig. 2A, C).

***Abdominal somites, telson, and uropods*.** Sixth abdominal somite 0.36–0.4 (median 0.38) ×length of carapace, 1.36–1.69 (median 1.45) × as long as fifth abdominal somite, 0.64–0.84 (median 0.73) × length of telson. Telson length 2.14–2.55 (median 2.27) × as long as proximal wide, distal margin triangular, terminating in a short median projection, with 4–6 pairs of dorsal spiniform setae and one pair of dorso-subdistal spiniform setae; distal end with three or four pairs of spiniform setae, lateral pair longer than intermediate pairs (Fig. 2D, E). Preanal carina low, slightly bent backwards, with few setae, lacking a spine (Fig. 2F). Uropodal diaeresis with 16–21 (median 20) movable spiniform setae, outermost longer than lateral angle (Fig. 2G).

***Mouthparts and branchiae*.** Incisor process of mandible ending in one row of 5–7 irregular teeth, molar process truncated (Fig. 2H). Lower lacinia of maxillula broadly rounded, upper lacinia elongated, with several distinct teeth and setae on inner margin, palp stout with few simple setae at tip (Fig. 2I). Upper endites of maxilla subdivided, palp short, scaphognathite tapering posteriorly, with numerous long, curved setae at posterior margin (Fig. 2J). Distal end of palp of first maxilliped triangular, with a short projection; flagellum of the exopod very elongated, endopod high, reaching 0.8 × length of flagellum of exopod (Fig. 2K, L). Podobranch of second maxilliped slightly reduced, with few finger-like projections (Fig. 2M). Third maxilliped reaching to end of antennular peduncle, ending in single terminal claw, exopod reaching 0.6 × length of penultimate segment; ultimate segment longer than penultimate segment; epipod present on the coxa (Fig. 2N). Branchial formula as typical for genus *Paracaridina* Liang, Guo & Tang, 1999, five pairs of pleurobranchs well developed; two pairs of arthrobranchs on third maxillipeds, with second pair strongly reduced in size; one pair of podobranchs on second maxilliped slightly reduced, arthrobranch on first pereiopod absent (Liang et al. 1999).

***Pereiopods*.** Epipods present on first fourth pereiopods. First pereiopod short, robust, reaching end of basal segment of antennular peduncle; chela 1.91–2.22 (median 2.02) × as long as wide, 1.22–1.44 (median 1.26) × length of carpus; tips of fingers rounded, without hook; dactylus 0.73–0.89 (median 0.83) × as long as palm; carpus excavated strongly anteriorly, 1.73–2.25 (median 1.89) × as long as wide; carpus 0.83–1.01 (median 0.95) × length of merus; merus 2.3–2.94 (median 2.67) × as long as wide, longer than ischium (Fig. 3A). Second pereiopod long, slender, reaching beyond distal end of antennular peduncle; chela 2.19–2.62 (median 2.4) × as long as wide, 0.60–0.78 (median 0.69) × length of carpus; tips of fingers rounded, without hook; dactylus 1.04–1.4 (median 1.17) × as long as palm; carpus 5.0–5.69 (median 5.16) × as long as wide, 0.93–1.05 (median 1.0) × as long as merus; merus 4.81–5.58 (median 5.12) × as long as wide, longer than ischium (Fig. 3B). Third pereiopod slender, reaching beyond distal end of antennular peduncle by its dactylus, terminating in one claw, with five accessory spiniform setae on flexor margin, dactylus 2.44–3.19 (median 2.82) × as long as wide (terminal claw and spiniform setae on flexor margin included), propodus 8.38–9.3 (median 8.79) × as long as wide, 4–4.64 (median 4.17) × as long as dactylus; carpus 3.41–4.41 (median 4.01) × as long as wide, 0.53–0.69 (median 0.62) × as long as propodus, 0.46–0.55 (median 0.52) × as long as merus; merus 5.33–6.68 (median 5.74) × as long as wide, bearing 3 strong, movable spiniform setae on posterior margin of outer surface; ischium with one small movable spiniform seta (Fig. 3C, D). Fifth pereiopod slender, reaching to end of third segment of antennular peduncle, dactylus 3.27–3.67 (median 3.53) × as long as wide (terminal claw and spiniform setae on flexor margin included), terminating in one large claw, with 30–37 spiniform setae on flexor margin; propodus 9.82–12.06 (median 10.55) × as long as wide, 3.06–3.98 (median 3.54) × length of dactylus; carpus 3.45–4.39 (median 4.0) × as long as wide, 0.46–0.57 (median 0.48) × as long as propodus, 0.56–0.66 (median 0.61) × as long as merus; merus 5.4–6.21 (median 5.99) × as long as wide, bearing 3 strong, movable spiniform setae on posterior margin of outer surface, ischium without movable spiniform setae (Fig. 3E, F).

***Pleopods*.** Endopod of male first pleopod extending to 0.62 × exopod, elongated and kidney-shaped, 2.43–3.04 (median 2.91) × as long as proximal width, inner margin concave, outer margin slightly convex, rounded distally, long pappose setae on outer and distal margins, medium-length setae on inner margin; with appendix interna curved upwards, exceeding terminal margin of endopod by 0.51 its length (Fig. 3G, H). Appendix masculina of male second pleopod slender, reaching to proximal 0.62 × endopod length, 7.38 × as long as distal width, stick-shaped, with some short spiniform setae on outer surface and some long spiniform setae on distal surface; appendix interna at the middle of appendix masculina, narrow, small, extending ~0.54 × length of appendix masculina (Fig. 3I, J).

##### Coloration.

**Figure 4.**
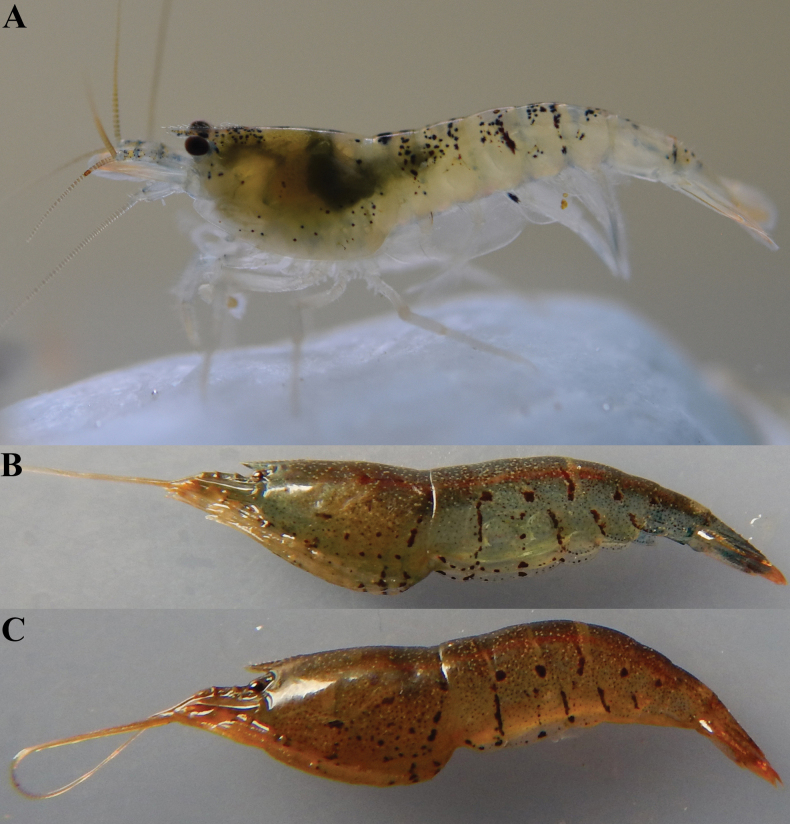
**A–C.** Variations in the of live coloration of *Caridinangocson* sp. nov. (ZMB 30276), collected in Hoa Binh Province, Vietnam.

The body is grey to slightly yellowish in color, with many small reddish black spots. There are several small stripes formed by spots on the abdominal part (Fig. 4A–C).

##### Reproductive biology.

One ovigerous female, ZMB 30276, cl 4.5 mm, developed eggs, with eyespots,1.33 × 0.84 mm (15 eggs).

##### Etymology.

The new species is named after the type locality, Ngoc Son Commune. The name is used as a noun in apposition.

##### Habitat.

**Figure 5.**
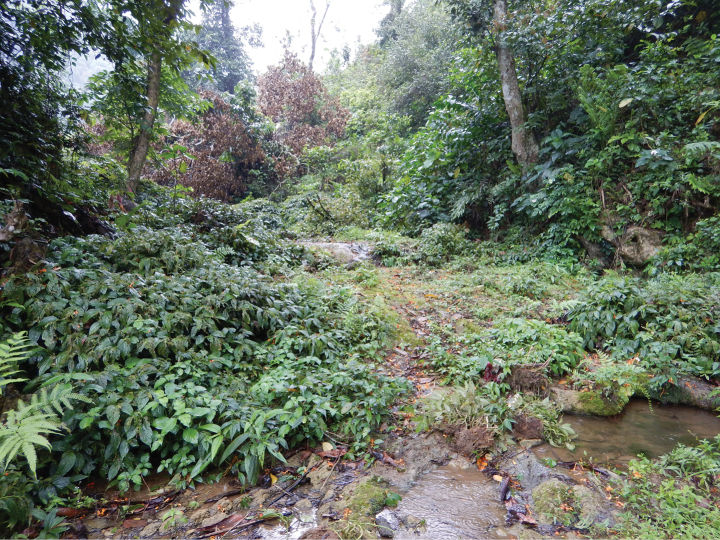
Habitat of *Caridinangocson* sp. nov. in Hoa Binh Province, Vietnam.

This new species was found in a stream with mixed sand, gravel, and rock substratum, and clear ﬂowing water from the forest (Fig. 5).

##### Distribution.

Our unpublished survey data throughout Vietnam indicate that this species is restricted to the Ngoc Son district of Hoa Binh province. The estimated area of occupancy is less than 500 km^2^. The altitude of the habitat area ranges from 200 to 700 m. The large eggs are also clearly indicative of a landlocked life cycle.

##### Molecular phylogenetic results.

*Caridinangocson* sp. nov. is well-supported as the sister species to *C.cucphuongensis* (Fig. 18). The minimum genetic divergence (p-distance) to *C.cucphuongensis*) is 6.2% (COI) and 2.7% (16S), respectively (Suppl. materials 1, 2).

##### Remarks.

*Caridinangocson* sp. nov. can be included in the *C.serrata* group based on a short rostrum, a long stylocerite that extends beyond the distal end of the basal antennular peduncle, the presence of dorsal teeth on the carapace, and the male pleopod endopod with a distinctive appendix interna (Cai and Ng 1999). However, *C.ngocson* sp. nov. differs from *C.serrata* in: the rostrum is longer (reaching to the beginning to middle of the second segment of antennular peduncle vs nearly reaching to or slightly exceeding the end of the basal segment of antennular peduncle); number of ventral teeth of the rostrum is smaller (0–2 vs 0–6); scaphocerite is stouter (2.6 vs 3.0 × as long as wide); endopod of male first pleopod is more slender (2.9 vs 2.5 × as long as proximal width); egg size is bigger (1.33 × 0.84 mm vs 0.9–1.0 × 0.7–0.6 mm) (Fig. 2A, C, G, H; cf. Cai and Ng 1999: fig. 2A, C, K, J).

The new species somewhat resembles *Caridinacucphuongensis* Dang, 1980 in the shape of the male first endopod, the appendix masculine and appendix internal of the male second pleopod. However, *C.ngocson* sp. nov. can be separated from *C.cucphuongensis* by a longer rostrum, reaching to the beginning to middle of second segment of antennular peduncle (vs just overreaching eyes to beginning of second segment); higher number of teeth on the dorsal rostrum, armed with 4–6 teeth on carapace posterior to orbital margin and 5–11 teeth on rostrum anterior to orbital margin (vs 0–3 teeth on carapace posterior to orbital margin and 2–7 teeth on rostrum anterior to orbital margin); slender chela of second pereiopod, 2.4 × as long as wide (vs 2.78 × as long as wide); a longer stylocerite, reaching to beginning or to 0.4 × length of second segment of antennular peduncle (vs reaching to beginning of second segment of antennular peduncle); higher number of spiniform setae on flexor margin of the dactylus of fifth pereiopod, 30–37 (vs 24–52 spiniform setae on flexor margin) (Figs 2A, B, 3B, F; cf. Phan et al. 2021b: figs 2A, B, 3B, F).

*Caridinangocson* sp. nov. and the following two new species *C.xuanlien* sp. nov. and *C.tanson*, have the defining character of *Paracaridina* Liang, Guo & Tang, 1999 because of their branchial formula (5 pairs of pleurobranchs well developed; 2 pairs of arthrobranchs on third maxillipeds, with second pair strongly reduced in size; 1 pair of podobranchs on second maxilliped slightly reduced, arthrobranch on first pereiopod absent). However, this character is not unique to *Paracaridina* (von Rintelen et al. 2008) and genetically the new species does not cluster with *Paracaridina* but – albeit with weak support – rather with *Neocaridina*. We have thus chosen the conservative approach of tentatively assigning it to *Caridina* given the clear need of a taxonomic revision at the genus level for these three genera (see Do et al. 2021b).

#### ﻿ Caridina xuanlien sp. nov.

Taxon classificationAnimaliaDecapodaAtyidae

A50CBB9C-1CBD-5F73-9030-5EBBFC07B754

https://zoobank.org/F9643BB7-BB97-4608-8A30-2C73F37C2FE6

Figs 6 , 7 , 8

##### Type material.

***Holotype*.** • Adult male, cl 4.5 mm, IB-FS 008, Vietnam, Thanh Hoa Province, Thuong Xuan District, Yen Nhan Commune, a small stream in Xuan Lien National Park, 19°56'05.9"N, 105°07'30.6"E, 21 November 2021, collected by Van Tu Do. ***Paratypes*.** • 8 females, cl 4.0–4.8 mm, 11 males, cl 3.6–4.7 mm, 49 additional specimens, ZMB 32948, same data as holotype; male, cl 4.5 mm, female, cl. 4.6 mm, 107 additional specimens, ZMB 32944, Vietnam, Thanh Hoa Province, Thuong Xuan District, Van Xuan Commune, a small stream in Xuan Lien National Park, 19°55'15.0"N, 105°09'12.3"E, 24 October 2021, collected by Van Tu Do.

##### Comparative material.

*Caridinanguyeni* Li & Liang, 2002: • adult male, cl. 4.7 mm, ZMB 30280, Vietnam, Cao Bang Province, Ha Quang District, Truong Ha Commune, Pac Bo village, a small stream near Le Nin stream, 22°59'4"N, 106°2'53.7"E, 25 May 2017, collected by Van Tu Do.

##### Diagnosis.

**Figure 6.**
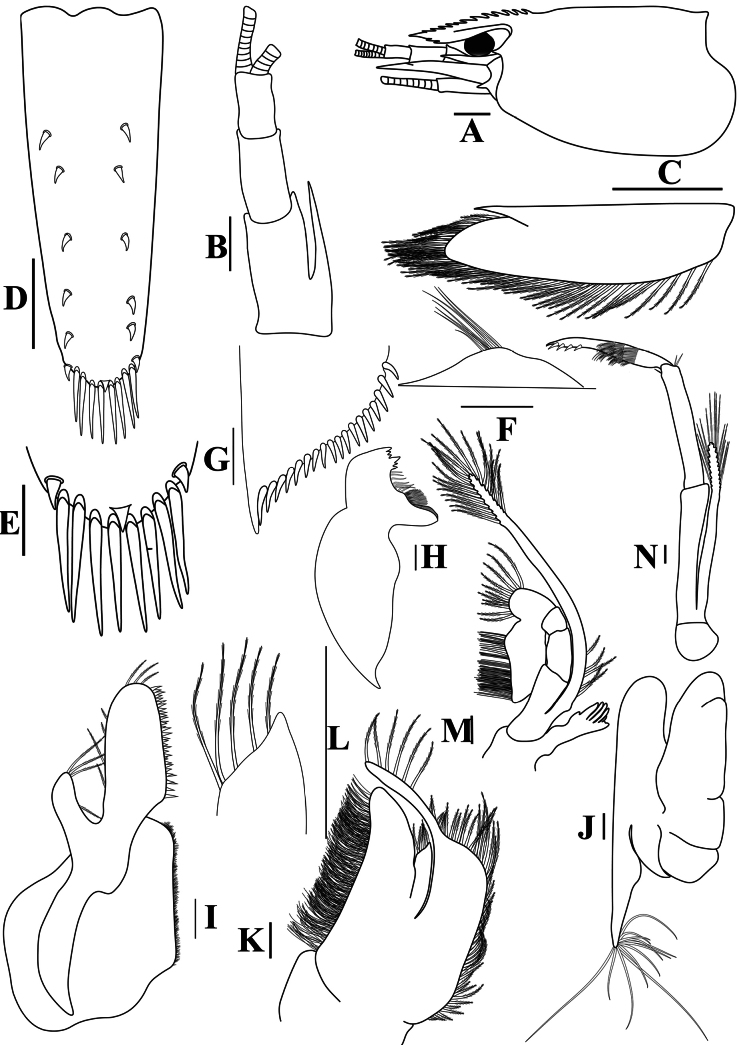
*Caridinaxuanlien* sp. nov. adult male, cl 4.5 mm (ZMB 32948). **A.** Cephalothorax and cephalic appendages, lateral view; **B.** Antennular peduncle; **C.** Scaphocerite; **D.** Telson; **E.** Distal portion of telson; **F.** Preanal carina; **G.** Uropodal diaeresis; **H.** Mandible; **I.** Maxillula; **J.** Maxilla; **K.** First maxilliped; **L.** Distal end of palp of first maxilliped; **M.** Second maxilliped; **N.** Third maxilliped. Scale bars: 1 mm (**A**); 0.5 mm (**B–D**); 0.2 mm (**E–K, M, N**); 0.1 mm (**L**).

**Figure 7.**
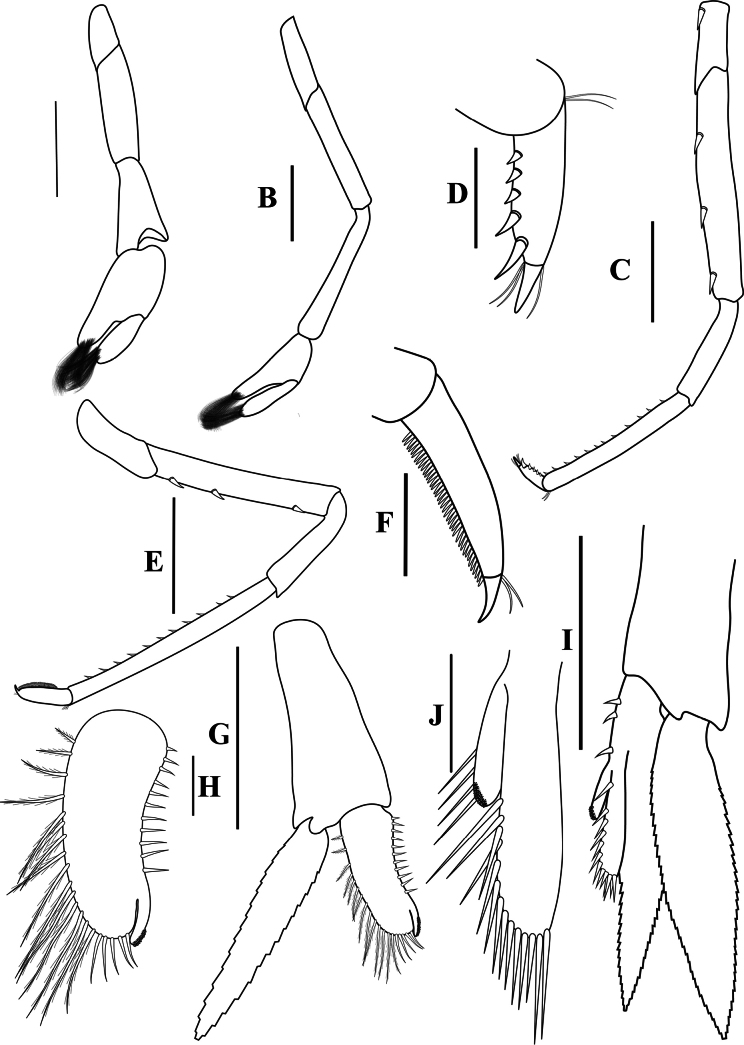
*Caridinaxuanlien* sp. nov. adult male, cl 4.5 mm (ZMB 32948). **A.** First pereiopod; **B.** Second pereiopod; **C.** Third pereiopod; **D.** The same, dactylus; **E.** Fifth pereiopod; **F.** The same, dactylus; **G.** Male first pleopod; **H.** Endopod of male first pleopod; **I.** Male second pleopod; **J.** Appendix masculina and interna of male second pleopod. Scale bars: 1 mm (**A, B, C, F, G, I**); 0.2 mm (**D, E, H, J**).

*Caridinaxuanlien* sp. nov. is characterized by several morphological characters such as short rostrum, just reaching to beginning of the third segment of antennular peduncle; stylocerite reaching to beginning of second segment of antennular peduncle (Fig. 6A, B); dactylus of first pereiopod longer than palm (Fig. 7A); short, kidney-shaped endopod of male first pleopod, extending to 2.79× exopod, with well-developed appendix interna, slightly exceeding terminal margin of endopod by 0.23 its length (Fig. 7G, H); short and stick-shaped appendix masculina of male second pleopod, reaching to proximal 0.43× endopod length, with narrow and small appendix interna, extending ~0.6× length of appendix masculina (Fig. 7I, J).

##### Description.

***Cephalothorax and cephalic appendages*.** Carapace length 3.6–4.8 mm (median 4.3 mm, *n* = 20). Rostrum short and slender, slightly downward, reaching to beginning of the third segment of antennular peduncle, 0.1–0.2 (median 0.2) × as long as carapace, rostral formula 2–7+3–9/0–2 (Fig. 6A). Suborbital angle acute, completely fused with antennal spine; pterygostomian margin rounded, slightly produced forward (Fig. 6A). Eyes well developed with globular cornea, anterior end reaching to 0.7× length of basal segment of antennular peduncle (Fig. 6A). Antennular peduncle 0.6–0.74 (median 0.64) × as long as carapace; basal segment 1.63–2.0 (median 1.88) × as long as second segment, second segment 1.17–1.4 (median 1.33) × as long as third segment (Fig. 6B). Stylocerite reaching to beginning of second segment of antennular peduncle (Fig. 6B). Scaphocerite elongated ovate, reaching beyond distal end of antennular peduncle, 2.68–2.88 (median 2.86) × as long as wide (Fig. 6A, C).

***Abdominal somites, telson, and uropods*.** Sixth abdominal somite 0.47–0.5 (median 0.47) × length of carapace, 1.47–1.62 (median 1.58) × as long as fifth abdominal somite, 0.85–0.95 (median 0.91) × length of telson. Telson 2.4–3.25 (median 2.75) × as long as proximal wide, distal margin triangular, terminating in a short median projection, with five or six pairs of dorsal spiniform setae and one pair of dorso-subdistal spiniform setae; distal end with three or four pairs of spiniform setae, lateral pair shorter than intermediate pairs (Fig. 6D, E). Preanal carina low,, with few setae, lacking a spine (Fig. 6F). Uropodal diaeresis with 18–21 (median 21) movable spiniform setae, outermost shorter than lateral angle (Fig. 6G).

***Mouthparts and branchiae*.** Incisor process of mandible ending in one row of six or seven irregular teeth, molar process truncated (Fig. 6H). Lower lacinia of maxillula broadly rounded, upper lacinia elongated, with a several distinct teeth and setae on inner margin, palp stout with few simple setae at tip (Fig. 6I). Upper endites of maxilla subdivided, palp short, scaphognathite tapering posteriorly, with numerous long, curved setae at posterior margin (Fig. 6J). Distal end of palp of first maxilliped triangular, with a short projection; flagellum of the exopod very elongated, endopod reaching 0.8 × length of flagellum of exopod (Fig. 6K, L). Podobranch of second maxilliped slightly reduced, with few finger-like projections (Fig. 6M). Third maxilliped reaching near the end of antennular peduncle, ending in single terminal claw, exopod reaching 0.3 × length of penultimate segment; ultimate segment slightly longer penultimate segment; epipod present on the coxa (Fig. 6N). Branchial formula as typical for *Paracaridina* genus, five pairs of pleurobranchs well developed; two pairs of arthrobranchs on third maxillipeds, with second pair strongly reduced in size; one pair of podobranchs on second maxillipeds slightly reduced, arthrobranch on first pereiopod absent (Liang et al. 1999).

***Pereiopods*.** Epipods present on first to fourth pereiopods. First pereiopod short, robust, reaching end of basal segment of antennular peduncle; chela 2.08–2.29 (median 2.12) × as long as wide, 1.21–1.47 (median 1.40) × length of carpus; tips of fingers rounded, without hook; dactylus longer than palm, 1.0–1.07 (median 1.03) × as long as palm; carpus excavated strongly anteriorly, 1.5–1.87 (median 1.73) × as long as, respectively; carpus 0.78–0.91 (median 0.84) × length of merus; merus 2.65–3.54 (median 3.33) × as long as wide, longer than ischium (Fig. 7A). Second pereiopod long, slender, reaching to distal end of antennular peduncle; chela 2.65–3.0 (median 2.86) × as long as wide, 0.66–0.74 (median 0.73) × length of carpus; tips of fingers rounded, without hook; dactylus 1.42–1.58 (median 1.46) × as long as palm; carpus 5.25–6.21 (median 5.6) × as long as wide, 1.05–1.21 (median 1.16) × as long as merus; merus 5.46–6.0 (median 5.71) × as long as wide, longer than ischium (Fig. 7B). Third pereiopod slender, reaching beyond distal end of antennular peduncle by its dactylus, terminating in one claw, with five accessory spiniform setae on flexor margin, dactylus 3.29–4 (median 3.57) × as long as wide (terminal claw and spiniform setae on flexor margin included), propodus 8.9–9.44 (median 9.0) × as long as wide, 3.24–4.0 (median 3.71) × as long as dactylus; carpus 4.2–4.92 (median 4.83) × as long as wide, 0.69–0.78 (median 0.72) × as long as propodus, 0.5–0.57 (median 0.56) × as long as merus; merus 5.75–6.82 (median 6.0) × as long as wide, bearing three strong, movable spiniform setae on posterior margin of outer surface; ischium with one small movable spiniform seta (Fig. 7C, D). Fifth pereiopod slender, reaching to end of third segment of antennular peduncle, dactylus 3.33–3.75 (median 3.5) × as long as wide (terminal claw and spiniform setae on flexor margin included), terminating in one large claw, with 37–40 spiniform setae on flexor margin; propodus 10.88–13.13 (median 11.88) × as long as wide, 3.22–3.53 (median 3.33) × length of dactylus; carpus 4.15–4.36 (median 4.28) × as long as wide, 0.52–0.55 (median 0.55) × as long as propodus, 0.6–0.69 (median 0.62) × as long as merus; merus 5.83–7.17 (median 6.57) × as long as wide, bearing 3 strong, movable spiniform setae on posterior margin of outer surface, ischium without movable spiniform setae (Fig. 7E, F).

***Pleopods*.** Endopod of male first pleopod extending to 0.59 × exopod, kidney-shaped, anterior part not folded backwards, 2.75–3.05 (median 2.79) × as long as proximal width, inner margin concave, outer margin slightly convex, rounded distally, long pappose setae on outer and distal margins, medium-length setae on inner margin; with appendix interna curved upwards, slightly exceeding terminal margin of endopod by 0.23 of its length (Fig. 7G, H). Appendix masculina of male second pleopod slender, reaching to proximal 0.6 × endopod length, 8.25× as long as distal width, stick-shaped, with some short spiniform setae on outer surface and some long spiniform setae on distal surface; appendix interna at the middle of appendix masculina, narrow, small, extending ~0.60 length of appendix masculina (Fig. 7I, J).

##### Coloration.

**Figure 8.**
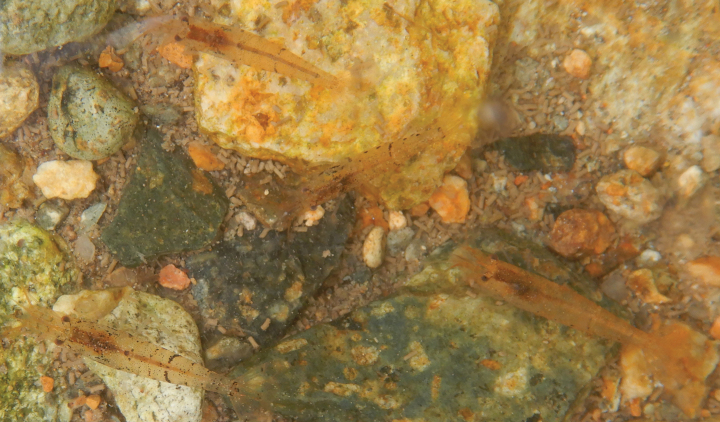
Live coloration of four specimens of *Caridinaxuanlien* sp. nov. (ZMB 32948), collected in Thanh Hoa Province, Vietnam.

The body is white to slightly yellowish in color, with many small black spots and small lines of irregular sizes (Fig. 8).

##### Etymology.

The new species is named after the type locality, Xuan Lien National Park. The name is used as a noun in apposition.

##### Habitat.

**Figure 9.**
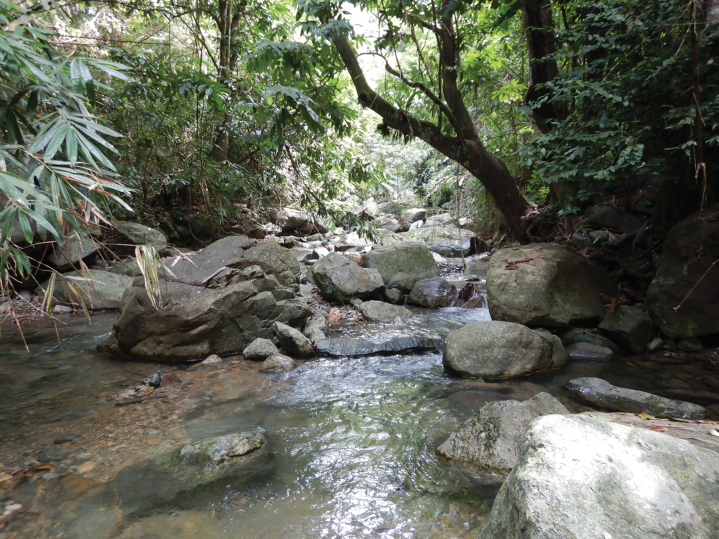
Habitat of *Caridinaxuanlien* sp. nov. (ZMB 32948) in Thanh Hoa Province, Vietnam.

This new species was found in the streams running in the forests. The substratum includes bedrock, stone, boulder, gravel, and sand in a water depth of 0.3–0.8 m (Fig. 9).

##### Distribution.

This species has been found only in the upland streams of Thuong Xuan District, Thanh Hoa Province, with elevations from 130 m to 600 m. The estimated area of occupancy is less than 1000 km^2^.

##### Molecular phylogenetic results.

*Caridinaxuanlien* sp. nov. is well supported as the sister species to *C.clinata* (Fig. 18). The minimum genetic divergence (p-distance) to *C.clinata*) is 4.0% (COI) and 1.5% (16S), respectively (Suppl. materials 1, 2).

##### Remarks.

The new species is quite similar to *C.nguyeni* from Cao Bang province, northeast Vietnam, in the shape of the rostrum and endopod of male first pleopod (Li and Liang 2002). However, it can be separated by the number of dorsal teeth on the rostrum anterior to the orbital margin (3–9 vs 9–12), the number of ventral teeth of the rostrum (0–2 vs 1–4); the length of stylocerite (reaching to the beginning of the second segment of antennular peduncle vs reaching to beyond the middle to the end of second segment of the antennular peduncle); the appendix interna of the second male pleopod (~0.6 of the length of the appendix masculina vs ~0.4 length of appendix masculina); the uropodal diaeresis (with 18–21 movable spiniform setae vs with 12–18 movable spiniform setae) (Figs 6A, B, 7G, H; cf. Li and Liang 2002, figs 1A, C, 2C, B).

The new species resembles *Caridinaclinata* Cai, Quynh & Ng, 1999 in the shape of the endopod of male first pleopod, appendix masculine, and appendix interna of male second pleopod. However, *C.xuanlien* sp. nov. can be separated from *C.clinata* by a longer rostrum, reaching to the beginning of third segment of antennular peduncle (vs not reaching distal margin of basal segment of antennular peduncle); fewer number of teeth on the rostrum, armed with 3–9 dorsal teeth on anterior to the orbital margin and 0–2 ventral teeth (vs 10–16 dorsal teeth on anterior to the orbital margin and 2–5 ventral teeth); a longer stylocertite, reaching beginning of second segment of antennular peduncle (vs not reaching to distal margin of basal segment of antennular peduncle); slender endopod of male first pleopod, 2.75× as long as proximal width (vs 2.5× as long as proximal width); higher number of movable spiniform setae on uropodal diaresis, 18–21 (vs 14–17 movable spiniform setae); higher number of spiniform setae on flexor margin of the dactylus of fifth pereiopod, 37–40 (vs 45–49 spiniform setae on flexor margin) (Figs 6A, B, G, 7H, F; cf. Cai et al. 1999: figs 1A, B, D, K, 2F).

#### ﻿ Caridina tanson sp. nov.

Taxon classificationAnimaliaDecapodaAtyidae

EE5C738F-2038-5400-A0C0-893854EE65A7

https://zoobank.org/E8C14EB4-078D-4544-82F5-E62DE2EB22B0

Figs 10 , 11 , 12

##### Type material.

***Holotype*.** • Adult male, cl 4.5 mm, IB-FS 009, Vietnam, Phu Tho Province, Tan Son District, Xuan Son Commune, Ban Lap Village, a small stream, 21°8'40.09"N, 104°56'40.739"E, 28 July 2022, collected by Thi Yen Phan. ***Paratypes*.** • 6 females, cl 4.0–6.0 mm, 10 males, cl 4.0–6.0 mm, ZMB 32979, same data as holotype; • male, cl 4.5 mm, ZMB 30729, Vietnam, Phu Tho Province, Tan Son District, Dong Son Commune, Than stream, site 2, 21°11'15.4"N, 104°52'22.9"E, 29 August 2013, collected by Tran Anh Duc.

##### Comparative material.

*Caridinapseudoserrata* Dang & Do, 2007, • adult male, cl 4.5 mm, ZMB 31570, Vietnam, Cao Bang Province, Phuc Hoa District, My Hung Commune, Na Rieng village, a stream runs inside Nguom Khuoi Khua Cave, 22°29'10.8"N, 106°33'3.27"E, 27 May 2017, collected by Van Tu Do.

##### Diagnosis.

**Figure 10.**
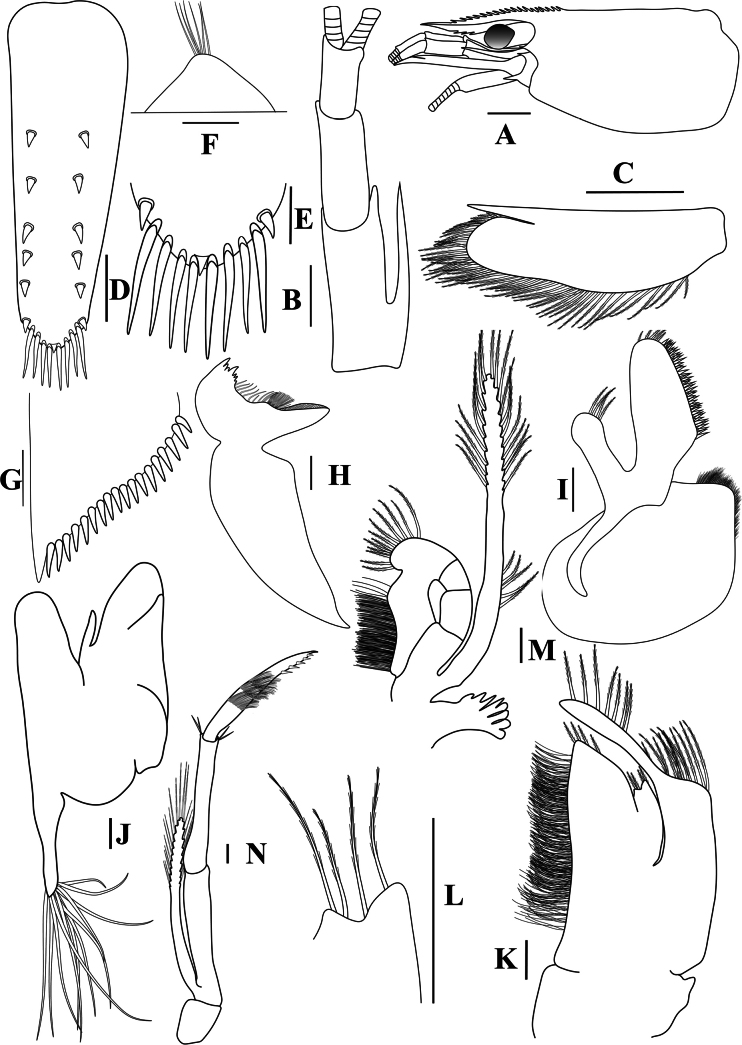
*Caridinatanson* sp. nov. adult male, cl 4.8 mm (ZMB 32979). **A.** Cephalothorax and cephalic appendages, lateral view; **B.** Antennular peduncle; **C.** Scaphocerite; **D.** Telson; **E.** Distal portion of telson; **F.** Preanal carina; **G.** Uropodal diaeresis; **H.** Mandible; **I.** Maxillula; **J.** Maxilla; **K.** First maxilliped; **L.** Distal end of palp of first maxilliped; **M.** Second maxilliped; **N.** Third maxilliped. Scale bars: 1 mm (**A**); 0.5 mm (**B–D**); 0.2 mm (**E–J**).

**Figure 11.**
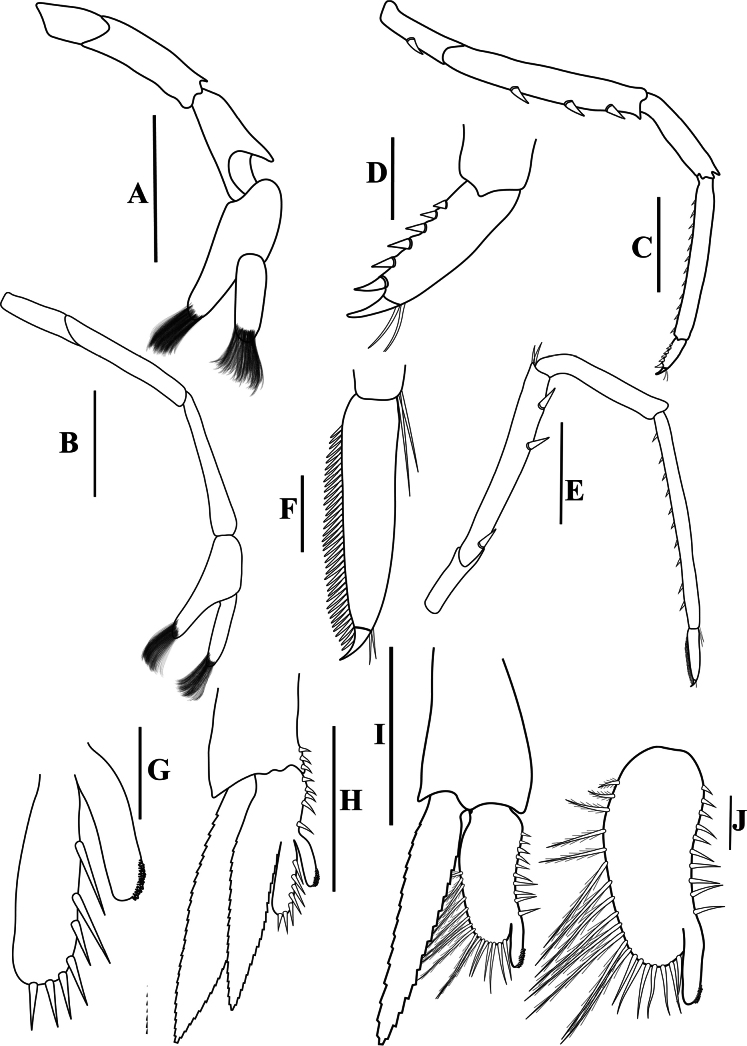
*Caridinatanson* sp. nov. adult male, cl 4.8 mm (ZMB 32979). **A.** First pereiopod; **B.** Second pereiopod; **C.** Third pereiopod; **D.** The same, dactylus; **E.** Fifth pereiopod; **F.** The same, dactylus; **G.** Male first pleopod; **H.** Endopod of male first pleopod; **I.** Male second pleopod; **J.** Appendix masculina and interna of male second pleopod. Scale bars: 1 mm (**A, B, C, F, G, I**); 0.2 mm (**D, E, H, J**).

This new species is characterized by rostrum short, straight, slender, tapering towards tip, reaching from beginning to end of third segment of antennular peduncle (Fig. 10A); stylocerite reaches from beginning to 0.2 × length of second segment of the antennular peduncle (Fig. 10B); carpus of fifth pereiopod slender, 7.3 × as long as wide (Fig. 11A); endopod of male first pleopod kidney-shaped, extending to 0.52 of exopod, 2.4 × as long as proximal width (Fig. 11G, H).

##### Description.

***Cephalothorax and cephalic appendages*.** Carapace length 4.0–6.4 mm (median 4.5 mm, *n* = 11). Rostrum slender, straight, reaching to beginning, sometime to the end of the third segment of antennular peduncle to distal end of antennular peduncle, 0.45–0.75 (median 0.52) as long as carapace, rostral formula 4–6+9–14/2–5 (Fig. 10A). Suborbital angle acute, completely fused with antennal spine; pterygostomian margin rounded, slightly produced forward (Fig. 10A). Eyes well developed with globular cornea, anterior end reaching to 0.7 length of basal segment of antennular peduncle (Fig. 10A). Antennular peduncle 0.46–0.67 (median 0.56) as long as carapace; basal segment 1.0–1.67 (median 1.25) × as long as second segment, second segment 1.14–2 (median 1.58) × as long as third segment (Fig. 10B). Stylocerite reaching to beginning or to 0.2 of length of second segment of antennular peduncle (Fig. 10B). Scaphocerite ovate, reaching beyond distal end of antennular peduncle, 3–3.81 (median 3.37) × as long as wide (Fig. 10A, C).

***Abdominal somites, telson, and uropods*.** Sixth abdominal somite 0.33–0.5 (median 0.4) length of carapace, 1.36–1.73 (median 1.5) × as long as fifth abdominal somite, 0.62–0.95 (median 0.76) length of telson. Telson length 2.0–2.89 (median 2.5) × as long as proximal wide, distal margin triangular, terminating in a short median projection, with 5–6 pairs of dorsal spiniform setae and one pair of dorso-subdistal spiniform setae; distal end with 4–5 pairs of spiniform setae, lateral pair longer than intermediate pairs (Fig. 10D, E). Preanal carina low, slightly bent backwards, with few setae, lacking a spine (Fig. 10F). Uropodal diaeresis with 16–21 (median 18) movable spiniform setae, outermost shorter than lateral angle (Fig. 10G).

***Mouthparts and branchiae*.** Incisor process of mandible ending in one row of 6–7 irregular teeth, molar process truncated (Fig. 10H). Lower lacinia of maxillula broadly rounded, upper lacinia elongated, with several distinct teeth and setae on inner margin, palp stout with few simple setae at tip (Fig. 11I). Upper endites of maxilla subdivided, palp short, scaphognathite tapering posteriorly, with numerous long, curved setae at posterior margin (Fig. 10J). Distal end of palp of first maxilliped triangular, with a short projection; flagellum of the exopod very elongated, endopod high, reaching 0.8 length of flagellum of exopod (Fig. 10K, L). Podobranch of second maxilliped slightly reduced, with few finger-like projections (Fig. 10M). Third maxilliped reaching to end of antennular peduncle, ending in single terminal claw, exopod reaching 0.4 length of penultimate segment; ultimate segment slightly longer than penultimate segment; epipod present on the coxa (Fig. 10N). Branchial formula as typical for *Paracaridina* genus, five pairs of pleurobranchs well developed; two pairs of arthrobranchs on third maxillipeds, with second pair strongly reduced in size; podobranch on second maxillipeds slightly reduced, arthrobranch on first pereiopod absent (Liang et al. 1999).

***Pereiopods*.** Epipods present on first to fourth pereiopods. First pereiopod short, robust, reaching end of basal segment of antennular peduncle; chela 1.91–2.75 (median 2.28) × as long as wide, 1.17–1.69 (median 1.41) × length of carpus; tips of fingers rounded, without hook; dactylus 0.77–1.18 (median 0.97) × as long as palm; carpus excavated strongly anteriorly, 1.6–2.29 (median 2.0) × as long as wide; carpus 0.79–1.0 (median 0.84) × length of merus; merus 2.5–3.6 (median 3.0) × as long as wide, longer than ischium (Fig. 11A). Second pereiopod long, slender, reaching beyond distal end of antennular peduncle; chela 2.11–3.63 (median 2.75) × as long as wide, 0.63–0.87 (median 0.76) × length of carpus; tips of fingers rounded, without hook; dactylus 0.92–1.4 (median 1.29) × as long as palm; carpus 4.6–6.0 (median 5.0) × as long as wide, 0.74–1.17 (median 1.0) × as long as merus; merus 4.8–7 (median 5.8) × as long as wide, longer than ischium (Fig. 11B). Third pereiopod slender, reaching beyond distal end of antennular peduncle by its dactylus, terminating in one claw, with five to six accessory spiniform setae on flexor margin, dactylus 2.67–4.0 (median 3.0) × as long as wide (terminal claw and spiniform setae on flexor margin included), propodus 9.75–12 (median 10.13) × as long as wide, 3.89–5.13 (median 4.44) × as long as dactylus; carpus 4–5.5 (median 4.5) × as long as wide, 0.49–0.69 (median 0.6) × as long as propodus, 0.43–0.56 (median 0.47) × as long as merus; merus 5.63–7.86 (median 7.01) × as long as wide, bearing three strong, movable spiniform setae on posterior margin of outer surface; ischium with one small movable spiniform seta (Fig. 11C, D). Fifth pereiopod slender, reaching to end of third segment of antennular peduncle, dactylus 3.67–7 (median 4.17) × as long as wide (terminal claw and spiniform setae on flexor margin included), terminating in one large claw, with 41–58 spiniform setae on flexor margin; propodus 10.75–14.67 (median 12.38) × as long as wide, 3.31–4.82 (median 4.01) × length of dactylus; carpus 4.17–6.25 (median 5) × as long as wide, 0.42–0.54 (median 0.48) × as long as propodus, 0.49–0.71 (median 0.58) × as long as merus; merus 6.5–8.6 (median 7.3) × as long as wide, bearing 3 strong, movable spiniform setae on posterior margin of outer surface, ischium without movable spiniform setae (Fig. 11E, F).

***Pleopods*.** Endopod of male first pleopod extending to 0.52 of exopod, elongated and kidney-shaped, 2.14–2.57 (median 2.43) × as long as proximal width, inner margin concave, outer margin slightly convex, rounded distally, long pappose setae on outer and distal margins, medium-length setae on inner margin; appendix interna long, exceeding terminal margin of endopod by 0.43 its length (Fig. 11G, H). Appendix masculina of male second pleopod slender, reaching to proximal 0.63 endopod length, 6.73 × as long as distal width, stick-shaped, with some short spiniform setae on outer surface and some long spiniform setae on distal surface; appendix interna at the middle of appendix masculina, small, extending ~0.66 length of appendix masculina (Fig. 11I, J).

##### Coloration.

**Figure 12.**
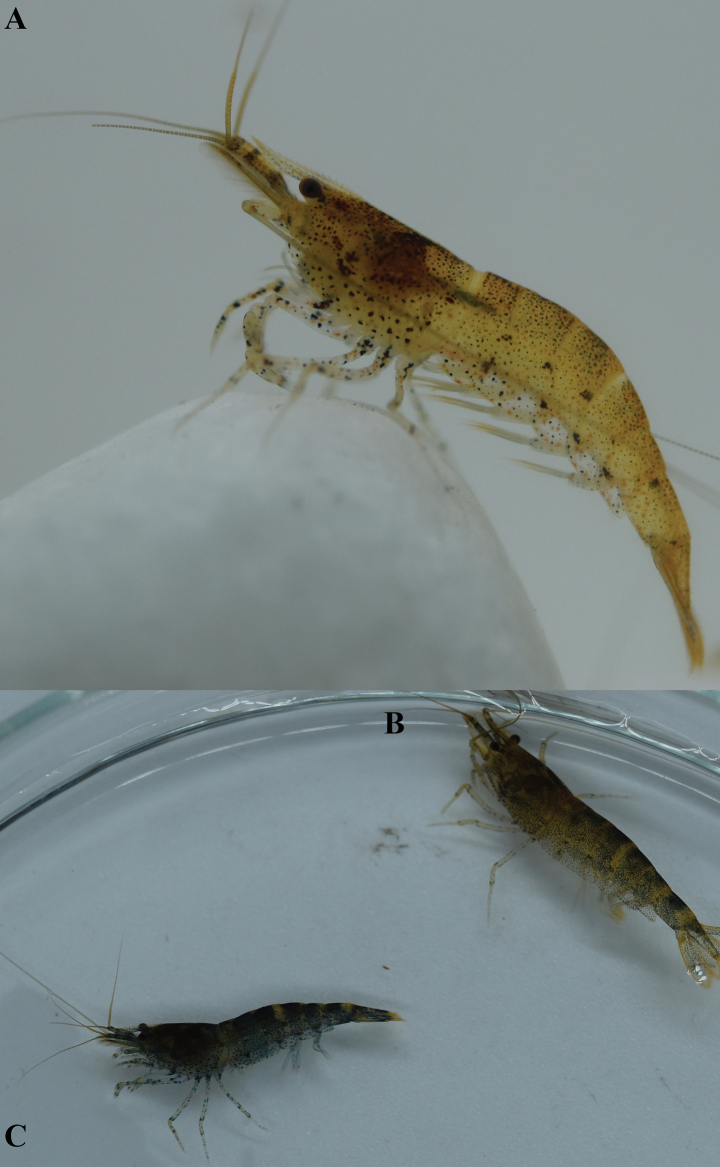
**A, B.** Live coloration of *Caridinatanson* sp. nov. (ZMB 32979), collected in Phu Tho Province, Vietnam.

The body is slightly yellowish to dark blue in color, with many small reddish spots and small lines of irregular sizes (Fig. 12A, B).

##### Reproductive biology.

Ovigerous female, ZMB 32979, cl 6.0 mm, eggs with eyespots 1.1 × 0.7 mm.

##### Etymology.

The new species is named after the type locality, Tan Son District. The name is used as a noun in apposition.

##### Habitat.

**Figure 13.**
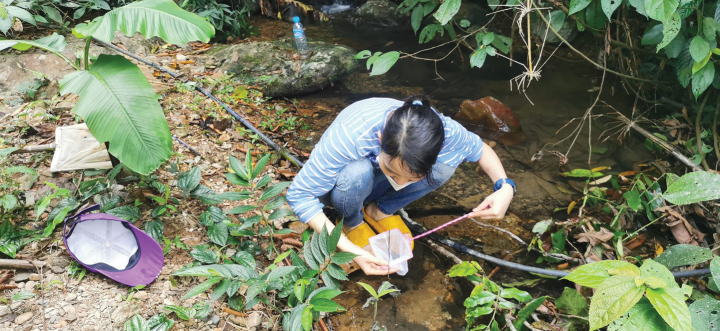
Collecting the samples in the habitat of *Caridinatanson* sp. nov. (ZMB 32979) in Phu Tho Province, Vietnam.

This new species was found in small streams. The substratum includes sand, gravel, bedrock, rotten leaves, and organic mulch in a water depth of 0.2–0.5 m (Fig. 13).

##### Distribution.

This species has only been recorded in the highland streams of Tan Son District, Phu Tho Province at altitudes from 200 m to 500 m. The estimated area of occupancy is less than 1000 km^2^.

##### Molecular phylogenetic results.

*Caridinatanson* sp. nov. is well supported as the sister group a clade comprising *C.haivanensis*, *C.clinata* and *C.xuanlien* sp. nov. (Fig. 18). The minimum genetic divergence (p-distance) to a species within that clade is 10.0% (COI, *C.haivanensis*) and 4.1% (16S, *C.clinata*), respectively (Suppl. materials 1, 2).

##### Remarks.

*Caridinatanson*, new species, is similar to *C.pseudoserrata*, also from northeast Vietnam, in the shape of the rostrum and endopod of male first pleopod (Dang and Do 2007a). The new species can be distinguished from *C.pseudoserrata* by a suite of characteristics: shorter stylocerite (reaching to the beginning or to 0.2 the length of the second segment of antennular peduncle vs reaching to 0.4–0.5 of second segment of antennular peduncle); shorter endopod of male first pleopod (2.4 × as long as proximal width vs 2.77 × as long as proximal width); longer appendix interna of endopod of male first pleopod (exceeding terminal margin of endopod by 0.43 its length vs not exceeding or just slightly exceeding the terminal margin of endopod) (Fig. 10A, B, 11 G, H; cf. Dang and Do 2007a: figs 1A, 2A, B).

*Caridinatanson* sp. nov. is also close to *C.xuanlien* sp. nov. in the shape of the rostrum and endopod of the male first pleopod. However, *C.tanson* can be distinguished from *C.xuanlien* sp. nov. by: there are more teeth on the rostrum, armed with 9–14 dorsal teeth on the rostrum anterior to the orbital margin and 2–5 ventral teeth (vs 3–9 dorsal teeth on the rostrum anterior to the orbital margin and 0–2 ventral teeth); the endopod of the male first pleopod is stouter, 2.43 × as long as proximal width (vs 2.79 × as long as proximal width); appendix interna of endopod of male first pleopod is longer, exceeding terminal margins of endopod by 0.43 of its length (vs exceeding terminal margins of endopod by 0.23 of its length); appendix masculina of male second pleopod is stouter, 6.73 × as long as distal width (vs 8.25 × as long as distal width).

#### ﻿ Caridina tamkim sp. nov.

Taxon classificationAnimaliaDecapodaAtyidae

0EBC4DD5-6620-5EF6-81C0-5C4E0995CC2C

https://zoobank.org/9C394B48-A399-4776-B693-7FF7F4B06702

**Figure 14.**
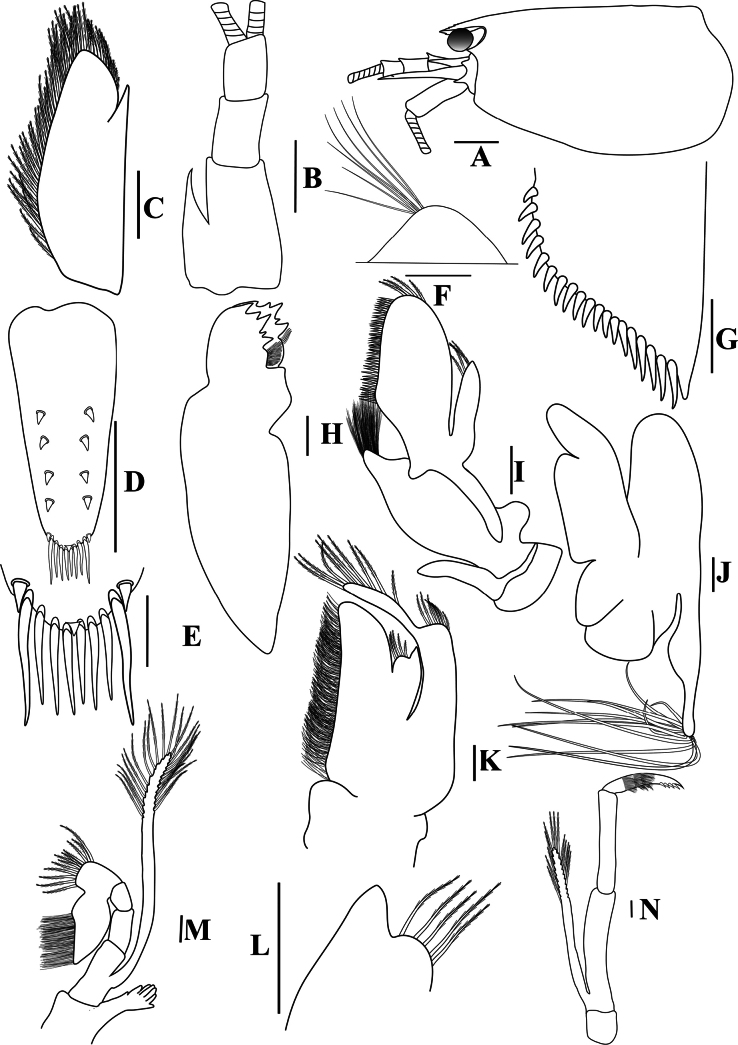
*Caridinatamkim* sp. nov. adult male, cl 5.2 mm (ZMB 32924). **A.** Cephalothorax and cephalic appendages, lateral view; **B.** Antennular peduncle; **C.** Scaphocerite; **D.** Telson; **E.** Distal portion of telson; **F.** Preanal carina; **G.** Uropodal diaeresis; **H.** Mandible; **I.** Maxillula; **J.** Maxilla; **K.** First maxilliped; **L.** Distal end of palp of first maxilliped; **M.** Second maxilliped; **N.** Third maxilliped. Scale bars: 1 mm (**A**); 0.5 mm (**B–D**); 0.2 mm (**E–K, M, N**); 0.1 mm (**L**).

**Figure 15.**
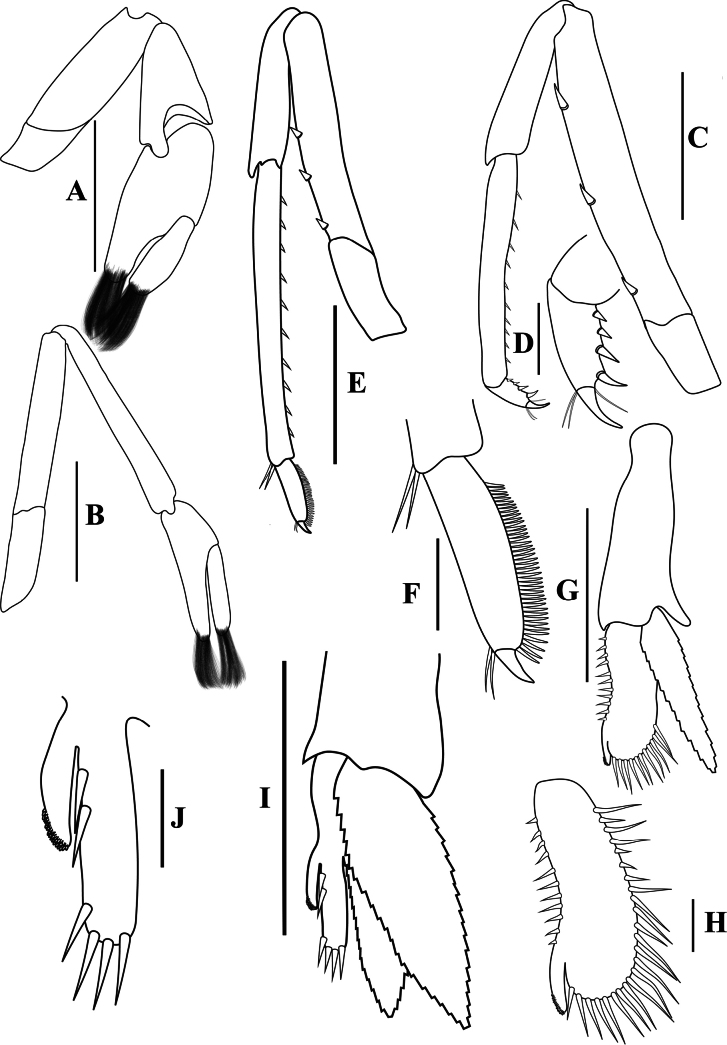
*Caridinatamkim* sp. nov. adult male, cl 5.2 mm (ZMB 32924). **A.** First pereiopod; **B.** Second pereiopod; **C.** Third pereiopod; **D.** The same, dactylus; **E.** Fifth pereiopod; **F.** The same, dactylus; **G.** Male first pleopod; **H.** Endopod of male first pleopod; **I.** Male second pleopod; **J.** Appendix masculina and interna of male second pleopod. Scale bars: 1 mm (**A, B, C, F, G, I**); 0.2 mm (**D, E, H, J**).

**Figure 16.**
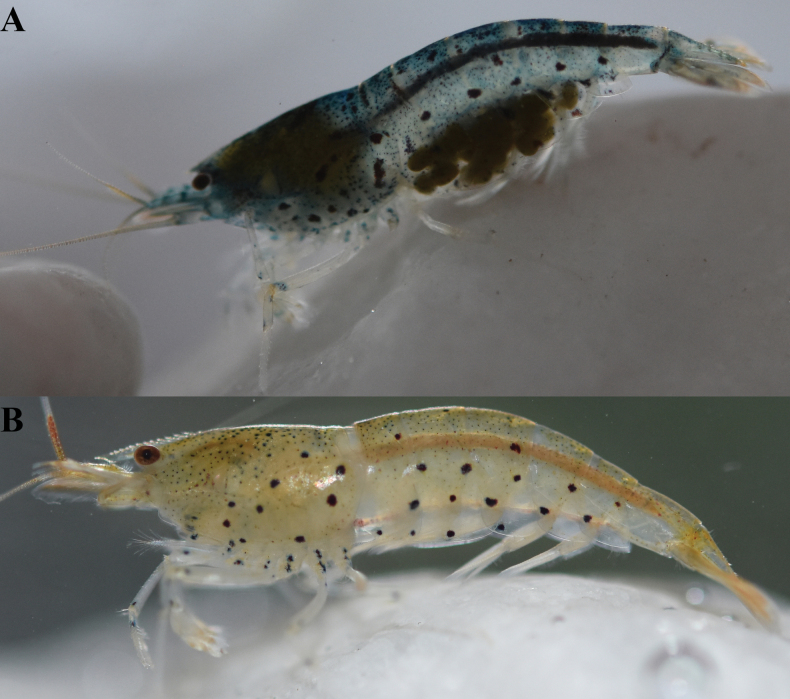
Live coloration of *Caridinatamkim* sp. nov. **A.**ZMB 32924; **B.**ZMB 33814, collected in Cao Bang Province, Vietnam.

**Figure 17.**
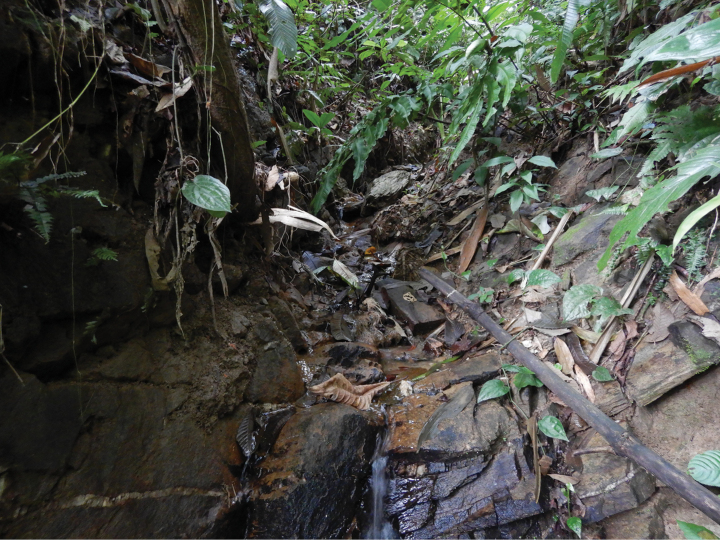
Habitat of *Caridinatamkim* sp. nov. (ZMB 33814) in Cao Bang Province, Vietnam.

Figs 14 , 15 , 16 , 17

##### Type material.

***Holotype*.** • Adult male, cl 4.8 mm, IB-FS 010, Vietnam, Cao Bang Province, Nguyen Binh District, Tam Kim Commune, a small stream, 22°34'53.207"N, 106°1'46.368"E, 08 October 2020, collected by Van Tu Do. ***Paratypes*.** • 14 females, cl 3.8–5.8 mm, 5 males, cl 4.0–5.4 mm, ZMB 32924, same data as holotype; • male, cl 4.5 mm, IB-FS 010_2, Vietnam, same location as holotype, 21 March 2024, collected by Van Tu Do; • two males, cl 4.4, 4.5 mm, female, cl 5.5 mm, 8 additional specimens, ZMB 32923, Vietnam, Nguyen Binh District, Tam Kim Commune, Tran Hung Dao forest, a well at the inflow of small stream, 22°35'37.902"N, 106°2'35.897"E, 08 October 2020, collected by Van Tu Do, Dang Van Dong; • 2 males, cl 4.3, 4.5 mm, 2 females, cl 5.0, 5.5 mm, 7 additional specimens, ZMB 33814, Vietnam, Cao Bang Province, Nguyen Binh District, a small stream near the road, 22°38'16.626"N, 105°58'32.135"E, collected by Van Tu Do, Dang Van Dong, Nguyen Tong Cuong, 5 October 2020; • male, cl 4.7 mm, female, cl 4.4 mm, 4 additional specimens, ZMB 33793, Vietnam, Cao Bang Province, Nguyen Binh District, Quang Thanh Commune, a small stream run out from Ong cave, 22°34'35.316"N, 105°55'25.998"E, collected by Van Tu Do, 7 October 2020; • male, cl 5.4 mm, female, cl 5.2 mm, 10 additional specimens, ZMB 33788, Vietnam, Cao Bang Province, Nguyen Binh District, a small stream near the road, 22°38'20.82"N, 105° 58'33.318"E, collected by Van Tu Do, 8 October 2020.

##### Comparative material.

*Caridinapacbo* Do, von Rintelen & Dang, 2020: • adult male, cl 4.2 mm, IB–FS 003 (Holotype), Vietnam, Cao Bang Province, Ha Quang District, Truong Ha Commune, Pac Bo Village, small stream in Khuoi Nam, 22°59'1.7"N, 106°02'31.2"E, 25 May 2017, collected by Van Tu Do.

##### Diagnosis.

This new species is characterized by several morphological characters such as short and slender rostrum, reaching just to the end of the basal segment of antennular peduncle or sometimes to the middle of second segment, without or with a few small teeth (Fig. 14A); medium stylocerite, reaching to the end of the basal segment or sometimes to beginning of second segment of the antennular peduncle (Fig. 14B); dactylus of the first pereiopod is shorter than palm (Fig. 15A); long, sub-rectangular endopod of the male first pleopod, extending to 0.87 of the length of the exopod, with short appendix interna, not or slightly exceeding the terminal margin of the endopod (Fig. 15G, H); short and stick-shaped appendix masculina of male second pleopod, reaching to proximal 0.8 of the length of the endopod, with narrow and small appendix interna, extending ~0.6 of the length of appendix masculina (Fig. 15 I, J).

##### Description.

***Cephalothorax and cephalic appendages*.** Carapace length 3.8–5.8 mm (median 4.6 mm, *n* = 20). Rostrum very short and slender, straight, mostly reaching near the end of basal segment of antennular peduncle, sometimes to middle of second segment, 0.1–0.2 (median 0.2) as long as carapace, teeth small, rostral formula 0–4+0–8/0–2 (Fig. 14A). Suborbital angle acute, completely fused with antennal spine; pterygostomian margin rounded, slightly produced forward (Fig. 14A). Eyes normally developed with globular cornea, eyestalk short, anterior end reaching to 0.6 of length of basal segment of antennular peduncle (Fig. 14A). Antennular peduncle 0.37–0.44 (median 0.43) × as long as carapace; basal segment 1.6–2.2 (median 1.67) × as long as second segment, second segment 1.2–1.25 (median 1.25) × as long as third segment (Fig. 14B). Stylocerite reaching to end of basal segment or sometime to beginning of second segment of antennular peduncle (Fig. 14A, B). Scaphocerite ovate, reaching beyond distal end of antennular peduncle, 2.33–2.67 (median 2.5) × as long as wide (Fig. 14A, C).

***Abdominal somites, telson, and uropods*.** Sixth abdominal somite 0.3–0.42 (median 0.38) × length of carapace, 1.31–1.64 (median 1.42) × as long as fifth abdominal somite, 0.76–1.0 (median 0.89) × length of telson. Telson length 2.1–2.38 (median 2.25) × as long as proximal wide, distal margin triangular, terminating in a short median projection, with six pairs of dorsal spiniform setae and one pair of dorso-subdistal spiniform setae; distal end with three or four pairs of spiniform setae, lateral pair slightly shorter than intermediate pairs (Fig. 14D, E). Preanal carina moderately high, slightly bent backwards, with few setae, lacking a spine (Fig. 14F). Uropodal diaeresis with 17–20 (median 18) movable spiniform setae, outermost longer than lateral angle (Fig. 14G).

***Mouthparts and branchiae*.** Incisor process of mandible ending in one row of six or seven irregular teeth, molar process truncated (Fig. 14H). Lower lacinia of maxillula broadly rounded, upper lacinia elongated, with several distinct teeth and setae on inner margin, palp stout with few simple setae at tip (Fig. 14I). Upper endites of maxilla subdivided, palp short, scaphognathite tapering posteriorly, with numerous long, curved setae at posterior margin (Fig. 14J). Distal end of palp of first maxilliped triangular, with a short projection; flagellum of the exopod very elongated, endopod high, reaching 0.9 of length of flagellum of exopod (Fig. 14K, L). Podobranch of second maxilliped slightly reduced, with few finger-like projections (Fig. 14M). Third maxilliped reaching near the end of antennular peduncle, ending in single terminal claw, exopod reaching 0.4 of length of penultimate segment; ultimate segment shorter penultimate segment; epipod present on the coxa (Fig. 14N). Branchial formula as is typical for genus, five pairs of pleurobranchs well developed; three pairs of arthrobranchs, two on third maxillipeds, with second pair strongly reduced in size, one pair on first pereiopod; one pair of podobranchs on second maxillipeds slightly reduced.

***Pereiopods*.** Epipods present on first to fourth pereiopods. First pereiopod short, robust, reaching beyond end of basal segment of antennular peduncle; chela 2.03–2.35 (median 2.11) × as long as wide, 1.37–1.5 (median 1.4) × length of carpus; tips of fingers rounded, with hook; dactylus shorter than palm, 0.82–0.9 (median 0.88) × as long as palm; carpus excavated strongly anteriorly, 1.5–1.75 (median 1.65) × as long as wide; carpus 0.8–1.0 (median 0.9) × length of merus; merus 2.39–2.67 (median 2.47) × as long as wide, longer than ischium (Fig. 15A). Second pereiopod long, slender, reaching to distal end of antennular peduncle; chela 3.16–3.5 (median 3.28) × as long as wide, 0.64–0.71 (median 0.67) × length of carpus; tips of fingers rounded, without hook; dactylus 1.33–1.64 (median 1.52) × as long as palm; carpus 6.07–7.08 (median 6.57) × as long as wide, 1.02–1.12 (median 1.11) × as long as merus; merus 5.92–6.38 (median 6.33) × as long as wide, longer than ischium (Fig. 15B). Third pereiopod slender, reaching beyond end of scaphocerite by its dactylus, terminating in one claw, with four or five accessory spiniform setae on flexor margin, dactylus 3.0–3.83 (median 3.64) × as long as wide (terminal claw and spiniform setae on flexor margin included), propodus 8.5–9.75 (median 9.0) × as long as wide, 3.48–3.950 (median 3.86) × as long as dactylus; carpus 4.69–4.83 (median 4.77) × as long as wide, 0.67–0.76 (median 0.72) × as long as propodus, 0.52–0.58 (median 0.53) × as long as merus; merus 5.63–6.59 (median 6.05) × as long as wide, bearing 3 strong, movable spiniform setae on posterior margin of outer surface; ischium without movable spiniform seta (Fig. 15C, D). Fifth pereiopod slender, reaching to end of second segment of antennular peduncle, dactylus 2.67–3.38 (median 3.13) × as long as wide (terminal claw and spiniform setae on flexor margin included), terminating in one large claw, with 37–39 spiniform setae on flexor margin; propodus 11.25–13.14 (median 11.5) × as long as wide, 3.41–3.91 (median 3.75) × length of dactylus; carpus 4.73–5.0 (median 5.0) × as long as wide, 0.56–0.6 (median 0.58) × as long as propodus, 0.63–0.71 (median 0.67) × as long as merus; merus 5.5–6.33 (median 6.31) × as long as wide, bearing 3 strong, movable spiniform setae on posterior margin of outer surface, ischium without movable spiniform setae (Fig. 15E, F).

***Pleopods*.** Endopod of male first pleopod extending to 0.87 of exopod, sub-rectangular in shape, anterior part not folded backwards, 3.15–3.35 (median 3.19) × as long as proximal width, inner margin concave, outer margin slightly convex, rounded distally, long simple setae on outer and distal margins, medium-length setae on inner margin; with appendix interna curved upwards, not or slightly exceeding terminal margin of endopod by 0.23 of its length (Fig. 15G, H). Appendix masculina of male second pleopod slender, reaching to proximal 0.8 of endopod length, 7.3 × as long as distal width, stick-shaped, with some spiniform setae on outer surface and on distal surface; appendix interna at the middle of appendix masculina, narrow, small, extending ~0.54 length of appendix masculina (Fig. 15I, J).

##### Coloration.

The body is slightly yellowish to dark blue in color, with many black spots of irregular sizes (Fig. 16A, B).

##### Reproductive biology.

Four ovigerous females, ZMB 33814, cl 5.0 mm, undeveloped eggs 1.1 × 0.7 mm, cl 5.5 mm, eggs with eyespots 1.1 mm × 0.8 mm.

##### Variation.

There are variations between the populations of the new species on the length of the rostrum, stylocerite, and number of teeth on rostrum. In general, compared to the specimens from the type locality and the nearby locality (ca 3 km, ZMB 32923) with the specimens from ZMB 33814, ZMB 33793, and ZMB 33788 they showed shorter rostrum (can reach only to the end of the basal segment of antennular peduncle vs reaching to the middle of the second segment of the antennular peduncle), lower number of teeth on the rostrum dorsal (up to 8 teeth vs up to 12 teeth) and shorter stylocerite (reaching to the end of the basal segment of the antennular peduncle vs reaching to the beginning of the second segment).

##### Etymology.

The new species is named after the type locality, Tam Kim Commune. The name is used as a noun in apposition.

##### Habitat.

This new species was found in small streams and pools running through the forest. The substratum includes sand, gravel, and bedrock in a water depth of 0.2–0.4 m (Fig. 17).

##### Distribution.

This species is only distributed in Nguyen Binh District, Cao Bang Province in the high elevation from 500 m to 800 m. The estimated area of occupancy is less than 1000 km^2^.

##### Molecular phylogenetic results.

**Figure 18.**
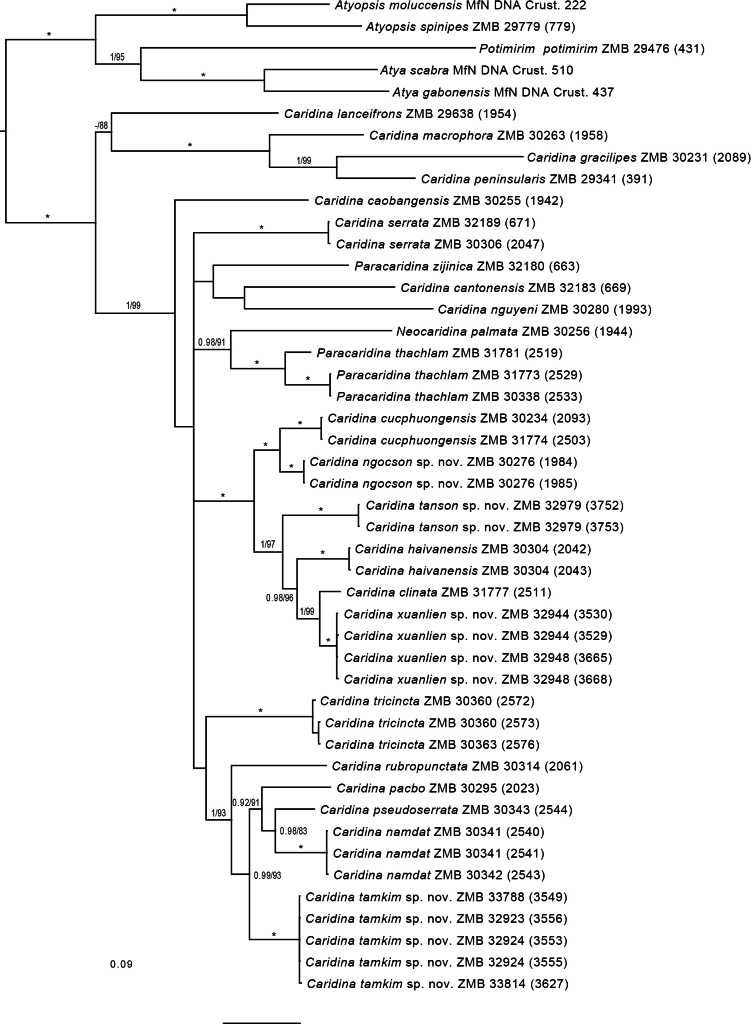
Bayesian Inference phylogram showing the relationships of the four new species of *Caridina* to other Vietnamese atyids, based on the analysis of two mitochondrial gene fragments. Numbers on branches show Bayesian posterior probabilities (>0.7) and ML bootstrap values (>70). An asterisk (*) indicates nodes with full support (1/100) in both analyses. The scale bar indicates the substitution rate. Numbers in brackets give the DNA accession number at MfN (MfN DNA Crust). See Table 1 for information on the sequenced specimens.

*Caridinatamkim* sp. nov. is well supported as the sister group a clade comprising *C.namdat*, *C.pacbo* and *C.pseudoserrata*. (Fig. 18). The minimum genetic divergence (p-distance) to a species within that clade (*C.namdat*) is 9.5% (COI) and 3.9% (16S), respectively (Suppl. materials 1, 2).

##### Remarks.

*Caridinatamkim*, new species, looks similar to *C.pacbo* in the shape of the rostrum and the male first pleopod (Do et al. 2020). However, it can be distinguished by a suite of characteristics: the lower number of teeth in the rostrum (0–8+0–4/0–2 vs 5–12+2–6/0–3); stylocerite extending (mostly reaching to end of basal segment, sometimes to the beginning of second segment vs reaching to the middle of the second segment of the antennular peduncle); slender merus of the first pereiopod (2.39–2.67 × as long as wide vs 2.75–3.8 × as long as wide); slender chela and merus of second pereiopod (3.16–3.5 × as long as wide vs 2.67–3.09 × as long as wide, 5.92–6.38 × as long as wide vs 5.0–5.86 × as long as wide, respectively); the distal margins of the endopod of the male first pleopod (slightly extended on both left and right sides vs not extended) (Figs 14A, B, 15A, B, G, H; cf. Do et al. 2020: figs 5A, B, 6A, B, G, H).

## ﻿Discussion

Four new species of atyid shrimp were collected from four different provinces in northern Vietnam. The results of our research of atyid shrimps in Vietnam revealed that northern Vietnam has a high level of diversity and endemism of Atyidae. Each province in northeastern Vietnam has at least one of its own *Caridina* species (Dang et al. 1980; Cai et al. 1999; Li and Liang 2002; Dang and Do 2007a; Do et al. 2020):

Thanh Hoa: *C.xuanlien* sp. nov.; Ninh Binh: *C.cucphuongensis* Dang in Dang, Thai & Pham, 1980, *C.clinata* Cai, Quynh & Ng, 1999 and *C.thachlam*; Hoa Binh: *C.ngocson* sp. nov.; Phu Tho: *C.tanson* sp. nov.; Cao Bang: *C.caobangensis* Li & Liang, 2002, *C.nguyeni* Li & Liang, 2002, *C.pseudoserrata* Dang & Do, 2007, *C.pacbo* Do, von Rintelen, Dang, 2020 and *C.tamkim* sp. nov.; Thai Nguyen: *C.rubropunctata* Dang & Do, 2007; Ha Giang and Tuyen Quang: *C.tricincta* Do, von Rintelen, Dang, 2020.

Northern Vietnam is characterized by a varied topography and climate with huge limestone mountain ranges (Sterling et al. 2006). The sampling sites are all located in limestone mountainous areas. Karst areas have formed “islands within islands”, and these are known to contain high levels of endemism (Clements et al. 2006). Our study revealed that the distribution area of atyid species in Vietnam is very limited, usually less than 1000 km^2^. The low number of eggs with large size indicates that these shrimps are landlocked species (Dudgeon 1987). The species with large eggs are confined to freshwater throughout their life cycle and have an abbreviated larval development. All of these species are endemic to relatively small geographic regions; many species have been shown to be confined to single lakes or river systems (von Rintelen and Cai 2009; Klotz and von Rintelen 2013). Their low dispersal ability has led to subsequent speciation, resulting in sometimes species-rich clades (von Rintelen et al. 2012). The present study increases the number of atyid species known from Vietnam to 30. The next integrative taxonomic studies, including genetic analyses, are likely to increase the number of species from this area.

## Supplementary Material

XML Treatment for Caridina ngocson

XML Treatment for Caridina xuanlien

XML Treatment for Caridina tanson

XML Treatment for Caridina tamkim

## ﻿Additional information

### Use of AI

No use of AI was reported.

### Author ORCIDs

Van Tu Do https://orcid.org/0000-0003-2881-1529

Thi Yen Phan https://orcid.org/0000-0001-9628-7584

Kristina von Rintelen https://orcid.org/0000-0003-4167-3570

Hung Anh Le https://orcid.org/0009-0004-2756-2393

Thomas von Rintelen https://orcid.org/0000-0002-6253-3078

### Data availability

All of the data that support the findings of this study are available in the main text or Supplementary Information.

## Supplementary materials

Supplementary material 1 p-distance for COI for all the relevant species Data type pdf https://binary.pensoft.net/file/1382292This dataset is made available under the Open Database License (http://opendatacommons.org/licenses/odbl/1.0/). The Open Database License (ODbL) is a license agreement intended to allow users to freely share, modify, and use this Dataset while maintaining this same freedom for others, provided that the original source and author(s) are credited.Van Tu Do, Thi Yen Phan, Kristina von Rintelen, Hung Anh Le, Thomas von Rintelen

Supplementary material 2 p-distance for 16S for all the relevant species Data type pdf https://binary.pensoft.net/file/1382293This dataset is made available under the Open Database License (http://opendatacommons.org/licenses/odbl/1.0/). The Open Database License (ODbL) is a license agreement intended to allow users to freely share, modify, and use this Dataset while maintaining this same freedom for others, provided that the original source and author(s) are credited.Van Tu Do, Thi Yen Phan, Kristina von Rintelen, Hung Anh Le, Thomas von Rintelen
