# Supplementary material for: ﻿Descriptions of four new species of atyid shrimp (Crustacea, Decapoda, Atyidae) in Vietnam
Source: Zookeys. 2025 Jul 28;1247:151–86. doi: 10.3897/zookeys.1247.148607 (PMC12322682; doi:10.3897/zookeys.1247.148607)
Supplement: Supplementary material 1 — p-distance for COI for all the relevant species [file zookeys-1247-151_article-148607__-s001.pdf]

ID	Species/sample	1	2	3	4	5	6	7	8	9	10	11	12	13	14	15	16	17	18	19	20	21	22	23	24	25
1	Caridina_cantonensis_ZMB_32183_1_CN																									
2	Caridina_caobangensis_ZMB_30255_1_VN	0.139																								
3	Caridina_clinata_ZMB_31777_1_VN	0.156	0.149																							
4	Caridina_cucphuongensis_ZMB_30234_1_VN	0.147	0.145	0.105																						
5	Caridina_cucphuongensis_ZMB_31774_1_VN	0.147	0.145	0.105	0.000																					
6	Caridina_gracilipes_ZMB_30231_1_VN	0.202	0.191	0.195	0.199	0.199																				
7	Caridina_haivanensis_ZMB_30304_1_VN	0.151	0.161	0.087	0.112	0.112	0.191																			
8	Caridina_haivanensis_ZMB_30304_2_VN	0.152	0.163	0.088	0.113	0.113	0.194	0.001																		
9	Caridina_lanceifrons_ZMB_29638_3_VN	0.172	0.166	0.169	0.171	0.171	0.191	0.176	0.175																	
10	Caridina_macrophora_ZMB_30263_1_VN	0.181	0.183	0.191	0.186	0.186	0.173	0.176	0.175	0.175																
11	Caridina_namdat_ZMB_30341_3_VN	0.140	0.130	0.140	0.140	0.140	0.184	0.139	0.141	0.178	0.162															
12	Caridina_namdat_ZMB_30341_4_VN	0.140	0.130	0.140	0.140	0.140	0.184	0.139	0.141	0.178	0.162	0.000														
13	Caridina_namdat_ZMB_30342_2_VN	0.140	0.130	0.140	0.140	0.140	0.184	0.139	0.141	0.178	0.162	0.000	0.000													
14	Caridina_ngocson_ZMB_30276_1_VN	0.132	0.135	0.110	**0.062**	**0.062**	0.196	0.102	0.104	0.169	0.160	0.122	0.122	0.122												
15	Caridina_ngocson_ZMB_30276_2_VN	0.132	0.135	0.110	**0.062**	**0.062**	0.196	0.102	0.104	0.169	0.160	0.122	0.122	0.122	0.000											
16	Caridina_nguyeni_ZMB_30280_2_VN	0.167	0.171	0.173	0.179	0.179	0.234	0.183	0.184	0.192	0.207	0.177	0.177	0.177	0.163	0.163										
17	Caridina_pacbo_ZMB_30295_2_VN	0.159	0.139	0.157	0.158	0.158	0.193	0.152	0.152	0.176	0.179	0.100	0.100	0.100	0.142	0.142	0.166									
18	Caridina_peninsularis_ZMB_29341_2_MY	0.175	0.197	0.193	0.197	0.197	0.155	0.188	0.191	0.198	0.149	0.187	0.187	0.187	0.183	0.183	0.207	0.211								
19	Caridina_pseudoserrata_ZMB_30343_1_VN	0.146	0.129	0.147	0.144	0.144	0.195	0.156	0.157	0.174	0.175	0.080	0.080	0.080	0.132	0.132	0.157	0.086	0.201							
20	Caridina_rubropunctata_ZMB_30314_1_VN	0.132	0.139	0.141	0.143	0.143	0.189	0.148	0.149	0.167	0.169	0.123	0.123	0.123	0.135	0.135	0.173	0.121	0.198	0.108						
21	Caridina_serrata_ZMB_30306_2_VN	0.130	0.133	0.158	0.141	0.141	0.196	0.149	0.150	0.173	0.164	0.133	0.133	0.133	0.136	0.136	0.179	0.147	0.195	0.135	0.140					
22	Caridina_serrata_ZMB_32189_1_CN	0.130	0.133	0.158	0.141	0.141	0.196	0.149	0.150	0.173	0.164	0.133	0.133	0.133	0.136	0.136	0.179	0.147	0.195	0.135	0.140	0.000				
23	Caridina_tamkim_ZMB_32923_1_VN	0.156	0.143	0.135	0.139	0.139	0.200	0.133	0.135	0.174	0.168	**0.095**	**0.095**	**0.095**	0.116	0.116	0.162	0.108	0.197	0.096	0.114	0.139	0.139			
24	Caridina_tamkim_ZMB_32924_1_VN	0.156	0.143	0.135	0.139	0.139	0.200	0.133	0.135	0.174	0.168	**0.095**	**0.095**	**0.095**	0.116	0.116	0.162	0.108	0.197	0.096	0.114	0.139	0.139	0.000		
25	Caridina_tamkim_ZMB_32924_2_VN	0.156	0.142	0.134	0.138	0.138	0.200	0.132	0.134	0.173	0.168	**0.095**	**0.095**	**0.095**	0.115	0.115	0.161	0.109	0.198	0.096	0.115	0.139	0.139	0.000	0.000	
26	Caridina_tamkim_ZMB_33788_1_VN	0.149	0.137	0.129	0.131	0.131	0.199	0.124	0.127	0.168	0.168	**0.096**	**0.096**	**0.096**	0.110	0.110	0.159	0.109	0.195	0.096	0.110	0.132	0.132	0.000	0.000	0.000
27	Caridina_tamkim_ZMB_33814_2_VN	0.157	0.144	0.134	0.140	0.140	0.200	0.132	0.134	0.174	0.171	**0.096**	**0.096**	**0.096**	0.118	0.118	0.163	0.111	0.199	0.099	0.117	0.141	0.141	0.002	0.002	0.002
28	Caridina_tanson_ZMB_32979_1_VN	0.153	0.157	0.110	0.114	0.114	0.199	**0.100**	**0.102**	0.179	0.173	0.141	0.141	0.141	0.107	0.107	0.178	0.148	0.206	0.151	0.153	0.138	0.138	0.145	0.145	0.144
29	Caridina_tanson_ZMB_32979_2_VN	0.153	0.157	0.110	0.114	0.114	0.199	**0.100**	**0.102**	0.179	0.173	0.141	0.141	0.141	0.107	0.107	0.178	0.148	0.206	0.151	0.153	0.138	0.138	0.145	0.145	0.144
30	Caridina_thachlam_ZMB_30338_2_VN	0.152	0.149	0.158	0.156	0.156	0.208	0.154	0.156	0.173	0.194	0.143	0.143	0.143	0.149	0.149	0.179	0.151	0.200	0.143	0.138	0.153	0.153	0.149	0.149	0.148
31	Caridina_thachlam_ZMB_31773_3_VN	0.152	0.149	0.158	0.156	0.156	0.208	0.154	0.156	0.173	0.194	0.143	0.143	0.143	0.149	0.149	0.179	0.151	0.200	0.143	0.138	0.153	0.153	0.149	0.149	0.148
32	Caridina_thachlam_ZMB_31781_2_VN	0.145	0.157	0.158	0.151	0.151	0.205	0.155	0.157	0.169	0.186	0.156	0.156	0.156	0.151	0.151	0.169	0.153	0.192	0.144	0.150	0.143	0.143	0.147	0.147	0.147
33	Caridina_tricincta_ZMB_30360_1_VN	0.156	0.152	0.162	0.152	0.152	0.199	0.157	0.158	0.191	0.183	0.135	0.135	0.135	0.141	0.141	0.175	0.136	0.191	0.140	0.136	0.146	0.146	0.135	0.135	0.136
34	Caridina_tricincta_ZMB_30360_2_VN	0.161	0.156	0.169	0.160	0.160	0.201	0.162	0.163	0.192	0.181	0.139	0.139	0.139	0.146	0.146	0.175	0.139	0.188	0.146	0.141	0.150	0.150	0.136	0.136	0.137
35	Caridina_tricincta_ZMB_30363_1_VN	0.158	0.157	0.171	0.158	0.158	0.202	0.161	0.162	0.194	0.180	0.139	0.139	0.139	0.145	0.145	0.178	0.141	0.187	0.147	0.143	0.149	0.149	0.138	0.138	0.138
36	Caridina_xuanlien_ZMB_32944_1	0.156	0.154	**0.041**	0.110	0.110	0.210	0.083	0.084	0.165	0.190	0.134	0.134	0.134	0.098	0.098	0.172	0.150	0.195	0.148	0.149	0.151	0.151	0.124	0.124	0.124
37	Caridina_xuanlien_ZMB_32944_2	0.161	0.158	**0.043**	0.110	0.110	0.213	0.084	0.085	0.169	0.197	0.141	0.141	0.141	0.104	0.104	0.180	0.159	0.196	0.157	0.155	0.156	0.156	0.135	0.135	0.134
38	Caridina_xuanlien_ZMB_32948_1_VN	0.156	0.156	**0.040**	0.110	0.110	0.212	0.083	0.084	0.166	0.193	0.139	0.139	0.139	0.101	0.101	0.173	0.155	0.195	0.153	0.153	0.153	0.153	0.129	0.129	0.128
39	Caridina_xuanlien_ZMB_32948_2	0.157	0.155	**0.041**	0.106	0.106	0.212	0.083	0.084	0.166	0.195	0.140	0.140	0.140	0.100	0.100	0.177	0.158	0.196	0.156	0.151	0.153	0.153	0.134	0.134	0.133
40	Neocaridina_palmata_ZMB_30256_1_VN	0.163	0.140	0.166	0.168	0.168	0.218	0.171	0.173	0.168	0.185	0.155	0.155	0.155	0.155	0.155	0.164	0.168	0.219	0.153	0.152	0.147	0.147	0.149	0.149	0.149
41	Paracaridina_zijinica_ZMB_32180_1_CN	0.148	0.127	0.160	0.142	0.142	0.204	0.153	0.154	0.162	0.198	0.142	0.142	0.142	0.137	0.137	0.168	0.146	0.195	0.139	0.140	0.137	0.137	0.150	0.150	0.149

**ID**	**Species/sample**	26	27	28	29	30	31	32	33	34	35	36	37	38	39	40
1	Caridina_cantonensis_ZMB_32183_1_CN															
2	Caridina_caobangensis_ZMB_30255_1_VN															
3	Caridina_clinata_ZMB_31777_1_VN															
4	Caridina_cucphuongensis_ZMB_30234_1_VN															
5	Caridina_cucphuongensis_ZMB_31774_1_VN															
6	Caridina_gracilipes_ZMB_30231_1_VN															
7	Caridina_haivanensis_ZMB_30304_1_VN															
8	Caridina_haivanensis_ZMB_30304_2_VN															
9	Caridina_lanceifrons_ZMB_29638_3_VN															
10	Caridina_macrophora_ZMB_30263_1_VN															
11	Caridina_namdat_ZMB_30341_3_VN															
12	Caridina_namdat_ZMB_30341_4_VN															
13	Caridina_namdat_ZMB_30342_2_VN															
14	**Caridina_ngocson_ZMB_30276_1_VN**															
15	**Caridina_ngocson_ZMB_30276_2_VN**															
16	Caridina_nguyeni_ZMB_30280_2_VN															
17	Caridina_pacbo_ZMB_30295_2_VN															
18	Caridina_peninsularis_ZMB_29341_2_MY															
19	Caridina_pseudoserrata_ZMB_30343_1_VN															
20	Caridina_rubropunctata_ZMB_30314_1_VN															
21	Caridina_serrata_ZMB_30306_2_VN															
22	Caridina_serrata_ZMB_32189_1_CN															
23	**Caridina_tamkim_ZMB_32923_1_VN**															
24	**Caridina_tamkim_ZMB_32924_1_VN**															
25	**Caridina_tamkim_ZMB_32924_2_VN**															
26	**Caridina_tamkim_ZMB_33788_1_VN**															
27	**Caridina_tamkim_ZMB_33814_2_VN**	0.003														
28	**Caridina_tanson_ZMB_32979_1_VN**	0.138	0.145													
29	**Caridina_tanson_ZMB_32979_2_VN**	0.138	0.145	0.000												
30	Caridina_thachlam_ZMB_30338_2_VN	0.147	0.150	0.163	0.163											
31	Caridina_thachlam_ZMB_31773_3_VN	0.147	0.150	0.163	0.163	0.000										
32	Caridina_thachlam_ZMB_31781_2_VN	0.145	0.149	0.155	0.155	0.076	0.076									
33	Caridina_tricincta_ZMB_30360_1_VN	0.133	0.138	0.158	0.158	0.171	0.171	0.167								
34	Caridina_tricincta_ZMB_30360_2_VN	0.135	0.139	0.164	0.164	0.172	0.172	0.169	0.012							
35	Caridina_tricincta_ZMB_30363_1_VN	0.136	0.140	0.163	0.163	0.169	0.169	0.169	0.012	0.002						
36	**Caridina_xuanlien_ZMB_32944_1_VN**	0.123	0.124	0.114	0.114	0.157	0.157	0.152	0.161	0.163	0.163					
37	**Caridina_xuanlien_ZMB_32944_2_VN**	0.123	0.134	0.114	0.114	0.160	0.160	0.155	0.168	0.172	0.172	0.000				
38	**Caridina_xuanlien_ZMB_32948_1_VN**	0.123	0.128	0.115	0.115	0.158	0.158	0.151	0.165	0.168	0.168	0.000	0.000			
39	**Caridina_xuanlien_ZMB_32948_2_VN**	0.123	0.133	0.111	0.111	0.158	0.158	0.153	0.164	0.168	0.168	0.000	0.004	0.000		
40	Caridina_zijinica_ZMB_32180_1_CN	0.144	0.149	0.161	0.161	0.155	0.155	0.145	0.151	0.158	0.158	0.152	0.157	0.155	0.153	
41	Neocaridina_palmata_ZMB_30256_1_VN	0.147	0.153	0.148	0.148	0.147	0.147	0.139	0.138	0.140	0.139	0.165	0.170	0.167	0.166	0.166
