# Supplementary material for: ﻿Descriptions of four new species of atyid shrimp (Crustacea, Decapoda, Atyidae) in Vietnam
Source: Zookeys. 2025 Jul 28;1247:151–86. doi: 10.3897/zookeys.1247.148607 (PMC12322682; doi:10.3897/zookeys.1247.148607)
Supplement: Supplementary material 2 — p-distance for 16S for all the relevant species [file zookeys-1247-151_article-148607__-s002.pdf]

ID	Species/samples	1	2	3	4	5	6	7	8	9	10	11	12	13	14	15	16	17	18	19	20	21
1	Caridina_cantonensis_ZMB_32183_1_CN																					
2	Caridina_caobangensis_ZMB_30255_1_VN	0.114																				
3	Caridina_clinata_ZMB_31777_1_VN	0.097	0.092																			
4	Caridina_cucphuongensis_ZMB_30234_1_VN	0.091	0.087	0.048																		
5	Caridina_cucphuongensis_ZMB_31774_1_VN	0.110	0.090	0.050	0.000																	
6	Caridina_gracilipes_ZMB_30231_1_VN	0.136	0.147	0.129	0.127	0.133																
7	Caridina_haivanensis_ZMB_30304_1_VN	0.101	0.096	0.030	0.062	0.066	0.137															
8	Caridina_haivanensis_ZMB_30304_2_VN	0.101	0.096	0.030	0.062	0.066	0.137	0.000														
9	Caridina_lanceifrons_ZMB_29638_3_VN	0.121	0.125	0.105	0.106	0.118	0.130	0.101	0.101													
10	Caridina_macrophora_ZMB_30263_1_VN	0.135	0.140	0.122	0.108	0.120	0.101	0.120	0.120	0.118												
11	Caridina_namdat_ZMB_30341_3_VN	0.084	0.086	0.069	0.081	0.082	0.144	0.071	0.071	0.108	0.127											
12	Caridina_namdat_ZMB_30341_4_VN	0.085	0.087	0.070	0.082	0.083	0.146	0.072	0.072	0.108	0.127	0.000										
13	Caridina_namdat_ZMB_30342_2_VN	0.082	0.088	0.071	0.079	0.080	0.146	0.073	0.073	0.110	0.129	0.002	0.002									
**14**	**Caridina_ngocson_ZMB_30276_1_VN**	0.107	0.093	0.041	**0.027**	**0.028**	0.133	0.054	0.054	0.110	0.120	0.069	0.070	0.071								
**15**	**Caridina_ngocson_ZMB_30276_2_VN**	0.107	0.093	0.041	**0.027**	**0.028**	0.133	0.054	0.054	0.110	0.120	0.069	0.070	0.071	0.000							
16	Caridina_nguyeni_ZMB_30280_2_VN	0.099	0.112	0.088	0.102	0.107	0.148	0.092	0.092	0.112	0.137	0.090	0.089	0.092	0.093	0.093						
17	Caridina_pacbo_ZMB_30295_2_VN	0.082	0.099	0.076	0.079	0.084	0.146	0.075	0.075	0.108	0.133	0.047	0.047	0.048	0.078	0.078	0.090					
18	Caridina_peninsularis_ZMB_29341_2_MY	0.114	0.138	0.121	0.108	0.118	0.088	0.122	0.122	0.123	0.073	0.125	0.127	0.127	0.120	0.120	0.131	0.125				
19	Caridina_pseudoserrata_ZMB_30343_1_VN	0.077	0.084	0.067	0.085	0.092	0.135	0.069	0.069	0.099	0.120	0.039	0.040	0.041	0.080	0.080	0.079	0.056	0.114			
20	Caridina_rubropunctata_ZMB_30314_1_VN	0.086	0.088	0.071	0.071	0.077	0.133	0.071	0.071	0.108	0.129	0.065	0.066	0.067	0.071	0.071	0.092	0.065	0.129	0.062		
21	Caridina_serrata_ZMB_30306_2_VN	0.084	0.095	0.071	0.077	0.086	0.146	0.075	0.075	0.120	0.137	0.077	0.078	0.079	0.082	0.082	0.114	0.088	0.135	0.069	0.058	
22	Caridina_serrata_ZMB_32189_1_CN	0.082	0.093	0.071	0.075	0.084	0.146	0.075	0.075	0.118	0.136	0.075	0.076	0.076	0.080	0.080	0.112	0.088	0.134	0.067	0.058	0.002
**23**	**Caridina_tamkim_ZMB_32923_1_VN**	0.080	0.088	0.063	0.079	0.079	0.138	0.065	0.065	0.103	0.133	**0.039**	**0.040**	**0.041**	0.069	0.069	0.079	0.043	0.118	0.037	0.043	0.073
**24**	**Caridina_tamkim_ZMB_32924_1_VN**	0.080	0.088	0.063	0.079	0.079	0.138	0.065	0.065	0.103	0.133	**0.039**	**0.040**	**0.041**	0.069	0.069	0.079	0.043	0.118	0.037	0.043	0.073
**25**	**Caridina_tamkim_ZMB_32924_2_VN**	0.080	0.088	0.063	0.079	0.079	0.138	0.065	0.065	0.103	0.133	**0.039**	**0.040**	**0.041**	0.069	0.069	0.079	0.043	0.118	0.037	0.043	0.073
**26**	**Caridina_tamkim_ZMB_33788_1_VN**	0.080	0.088	0.063	0.079	0.079	0.138	0.065	0.065	0.103	0.133	**0.039**	**0.040**	**0.041**	0.069	0.069	0.079	0.043	0.118	0.037	0.043	0.073
**27**	**Caridina_tamkim_ZMB_33814_2_VN**	0.080	0.088	0.063	0.079	0.079	0.138	0.065	0.065	0.103	0.133	**0.039**	**0.040**	**0.041**	0.069	0.069	0.079	0.043	0.118	0.037	0.043	0.073
**28**	**Caridina_tanson_ZMB_32979_1_VN**	0.095	0.097	**0.041**	0.062	0.065	0.144	0.054	0.054	0.121	0.133	0.077	0.078	0.079	0.058	0.058	0.090	0.079	0.129	0.077	0.067	0.069
**29**	**Caridina_tanson_ZMB_32979_2_VN**	0.095	0.097	**0.041**	0.062	0.065	0.144	0.054	0.054	0.121	0.133	0.077	0.078	0.079	0.058	0.058	0.090	0.079	0.129	0.077	0.067	0.069
30	Caridina_thachlam_ZMB_30338_2_VN	0.095	0.090	0.073	0.075	0.080	0.125	0.077	0.077	0.103	0.114	0.077	0.078	0.079	0.086	0.086	0.086	0.071	0.105	0.062	0.069	0.084
31	Caridina_thachlam_ZMB_31773_3_VN	0.094	0.088	0.071	0.073	0.079	0.124	0.075	0.075	0.101	0.113	0.075	0.076	0.077	0.084	0.084	0.084	0.069	0.103	0.062	0.068	0.082
32	Caridina_thachlam_ZMB_31781_2_VN	0.086	0.084	0.064	0.064	0.071	0.120	0.067	0.067	0.104	0.112	0.075	0.076	0.077	0.077	0.077	0.086	0.067	0.093	0.060	0.064	0.080
33	Caridina_tricincta_ZMB_30360_1_VN	0.095	0.086	0.069	0.079	0.084	0.123	0.073	0.073	0.110	0.120	0.060	0.061	0.062	0.075	0.075	0.088	0.067	0.112	0.052	0.067	0.079
34	Caridina_tricincta_ZMB_30360_2_VN	0.093	0.088	0.071	0.079	0.084	0.125	0.073	0.073	0.108	0.122	0.058	0.059	0.060	0.075	0.075	0.086	0.065	0.110	0.050	0.069	0.080
35	Caridina_tricincta_ZMB_30363_1_VN	0.093	0.088	0.071	0.079	0.084	0.125	0.073	0.073	0.108	0.122	0.058	0.059	0.060	0.075	0.075	0.086	0.065	0.110	0.050	0.069	0.080
**36**	**Caridina_xuanlien_ZMB_32944_1_VN**	0.094	0.090	**0.015**	0.050	0.052	0.129	0.030	0.030	0.101	0.120	0.066	0.066	0.067	0.045	0.045	0.084	0.073	0.118	0.064	0.069	0.067
**37**	**Caridina_xuanlien_ZMB_32944_2_VN**	0.094	0.090	**0.015**	0.050	0.052	0.129	0.030	0.030	0.101	0.120	0.066	0.066	0.067	0.045	0.045	0.084	0.073	0.118	0.064	0.069	0.067
**38**	**Caridina_xuanlien_ZMB_32948_1_VN**	0.094	0.090	**0.015**	0.050	0.052	0.129	0.030	0.030	0.101	0.120	0.066	0.066	0.067	0.045	0.045	0.084	0.073	0.118	0.064	0.069	0.067
**39**	**Caridina_xuanlien_ZMB_32948_2_VN**	0.096	0.092	**0.017**	0.052	0.054	0.127	0.028	0.028	0.103	0.118	0.067	0.068	0.069	0.047	0.047	0.086	0.075	0.116	0.066	0.071	0.069
40	Neocaridina_palmata_ZMB_30256_1_VN	0.105	0.116	0.095	0.095	0.105	0.133	0.097	0.097	0.114	0.125	0.090	0.091	0.088	0.107	0.107	0.112	0.099	0.116	0.082	0.081	0.097
41	Paracaridina_zijinica_ZMB_32180_1_CN	0.099	0.107	0.090	0.095	0.097	0.144	0.094	0.094	0.114	0.137	0.097	0.098	0.099	0.090	0.090	0.110	0.099	0.133	0.088	0.090	0.084

ID	Species/samples	22	23	24	25	26	27	28	29	30	31	32	33	34	35	36	37	38	39	40
1	Caridina_cantonensis_ZMB_32183_1_CN																			
2	Caridina_caobangensis_ZMB_30255_1_VN																			
3	Caridina_clinata_ZMB_31777_1_VN																			
4	Caridina_cucphuongensis_ZMB_30234_1_VN																			
5	Caridina_cucphuongensis_ZMB_31774_1_VN																			
6	Caridina_gracilipes_ZMB_30231_1_VN																			
7	Caridina_haivanensis_ZMB_30304_1_VN																			
8	Caridina_haivanensis_ZMB_30304_2_VN																			
9	Caridina_lanceifrons_ZMB_29638_3_VN																			
10	Caridina_macrophora_ZMB_30263_1_VN																			
11	Caridina_namdat_ZMB_30341_3_VN																			
12	Caridina_namdat_ZMB_30341_4_VN																			
13	Caridina_namdat_ZMB_30342_2_VN																			
**14**	Caridina_ngocson_ZMB_30276_1_VN																			
**15**	Caridina_ngocson_ZMB_30276_2_VN																			
16	Caridina_nguyeni_ZMB_30280_2_VN																			
17	Caridina_pacbo_ZMB_30295_2_VN																			
18	Caridina_peninsularis_ZMB_29341_2_MY																			
19	Caridina_pseudoserrata_ZMB_30343_1_VN																			
20	Caridina_rubropunctata_ZMB_30314_1_VN																			
21	Caridina_serrata_ZMB_30306_2_VN																			
22	Caridina_serrata_ZMB_32189_1_CN																			
**23**	Caridina_tamkim_ZMB_32923_1_VN	0.071																		
**24**	Caridina_tamkim_ZMB_32924_1_VN	0.071	0.000																	
**25**	Caridina_tamkim_ZMB_32924_2_VN	0.071	0.000	0.000																
**26**	Caridina_tamkim_ZMB_33788_1_VN	0.071	0.000	0.000	0.000															
**27**	Caridina_tamkim_ZMB_33814_2_VN	0.071	0.000	0.000	0.000	0.000														
**28**	Caridina_tanson_ZMB_32979_1_VN	0.069	0.073	0.073	0.073	0.073	0.073													
**29**	Caridina_tanson_ZMB_32979_2_VN	0.069	0.073	0.073	0.073	0.073	0.073	0.000												
30	Caridina_thachlam_ZMB_30338_2_VN	0.084	0.064	0.064	0.064	0.064	0.064	0.077	0.077											
31	Caridina_thachlam_ZMB_31773_3_VN	0.082	0.062	0.062	0.062	0.062	0.062	0.075	0.075	0.000										
32	Caridina_thachlam_ZMB_31781_2_VN	0.080	0.058	0.058	0.058	0.058	0.058	0.067	0.067	0.015	0.013									
33	Caridina_tricincta_ZMB_30360_1_VN	0.077	0.047	0.047	0.047	0.047	0.047	0.080	0.080	0.060	0.058	0.054								
34	Caridina_tricincta_ZMB_30360_2_VN	0.079	0.045	0.045	0.045	0.045	0.045	0.080	0.080	0.062	0.060	0.056	0.002							
35	Caridina_tricincta_ZMB_30363_1_VN	0.079	0.045	0.045	0.045	0.045	0.045	0.080	0.080	0.062	0.060	0.056	0.002	0.000						
**36**	Caridina_xuanlien_ZMB_32944_1_VN	0.067	0.060	0.060	0.060	0.060	0.060	0.034	0.034	0.071	0.069	0.062	0.067	0.067	0.067					
**37**	Caridina_xuanlien_ZMB_32944_2_VN	0.067	0.060	0.060	0.060	0.060	0.060	0.034	0.034	0.071	0.069	0.062	0.067	0.067	0.067	0.000				
**38**	Caridina_xuanlien_ZMB_32948_1_VN	0.067	0.060	0.060	0.060	0.060	0.060	0.034	0.034	0.071	0.069	0.062	0.067	0.067	0.067	0.000	0.000			
**39**	Caridina_xuanlien_ZMB_32948_2_VN	0.069	0.062	0.062	0.062	0.062	0.062	0.036	0.036	0.073	0.071	0.064	0.066	0.066	0.066	0.002	0.002	0.002		
40	Neocaridina_palmata_ZMB_30256_1_VN	0.097	0.082	0.082	0.082	0.082	0.082	0.107	0.107	0.073	0.071	0.069	0.084	0.086	0.086	0.094	0.094	0.094	0.096	
41	Paracaridina_zijinica_ZMB_32180_1_CN	0.084	0.084	0.084	0.084	0.084	0.084	0.094	0.094	0.079	0.077	0.079	0.088	0.090	0.090	0.086	0.086	0.086	0.088	0.105
